# Supplementary figures and images for: Evaluation of information flows in the RAS-MAPK system using transfer entropy measurements
Source: eLife. 2025 Mar 6;14:e104432. doi: 10.7554/eLife.104432 (PMC11884788; doi:10.7554/eLife.104432)

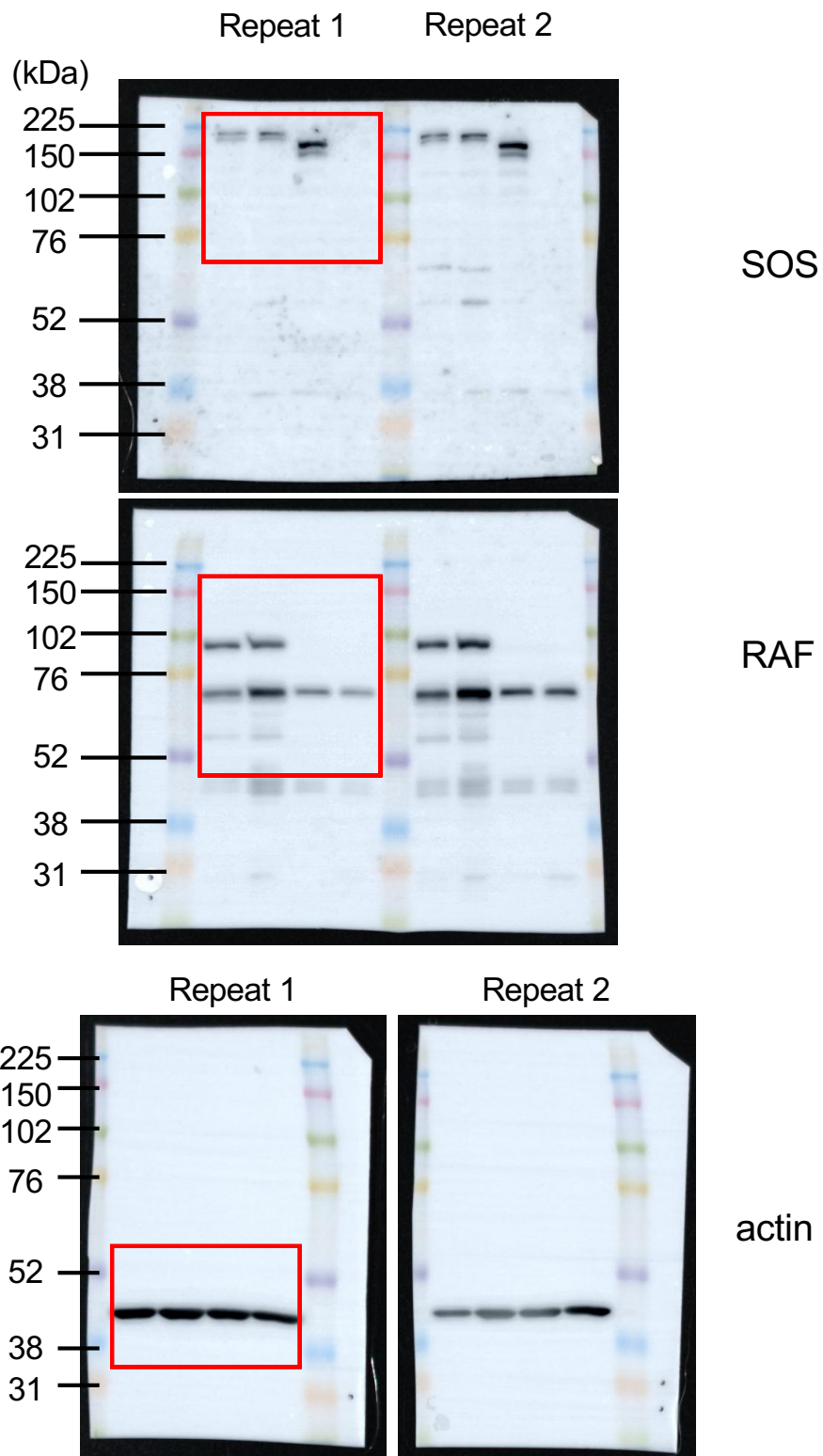

Figure 1a -source data 1

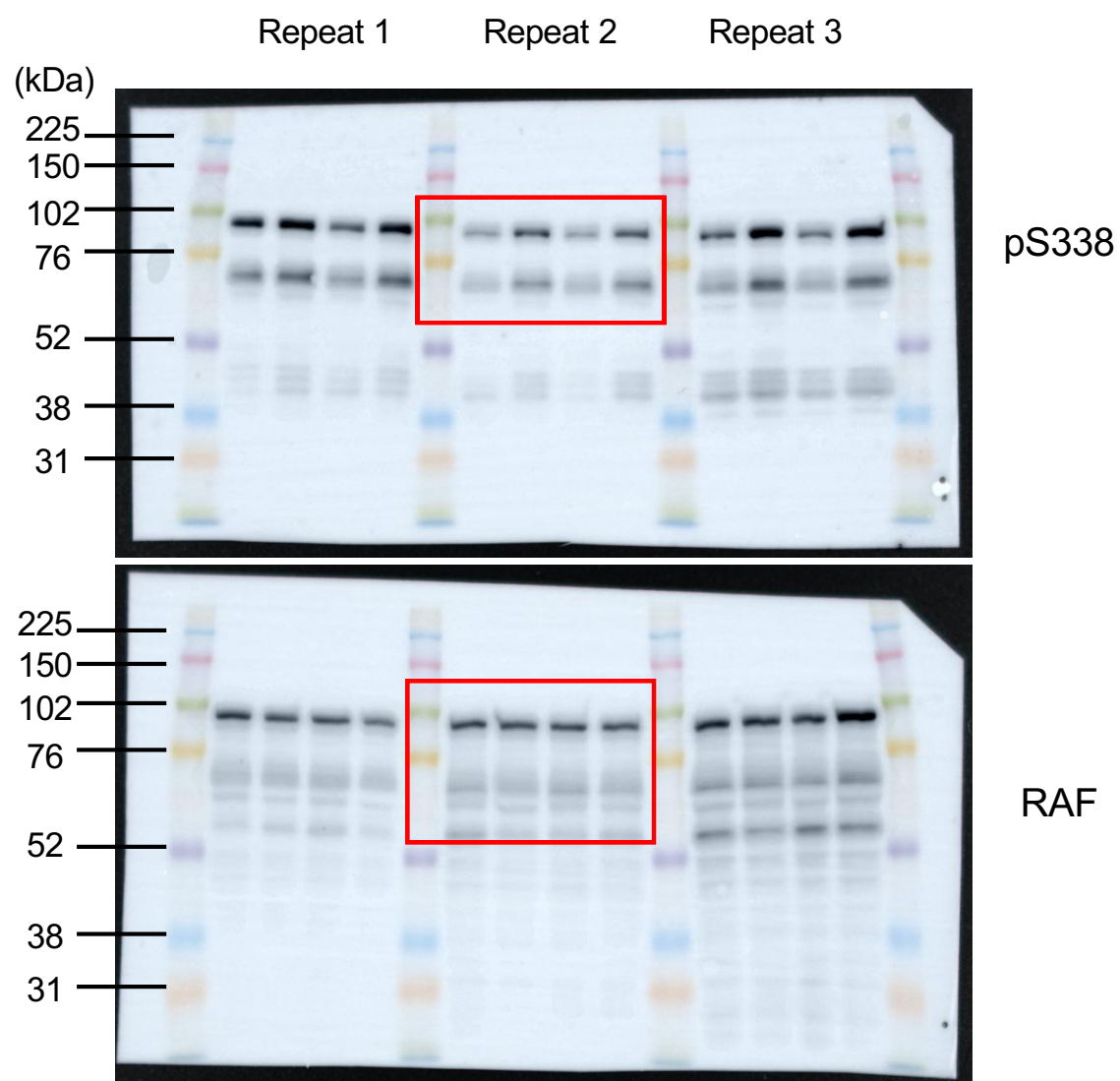

Figure 1b -source data 1

Supplement: Figure 1—source data 1. [file elife-104432-fig1-data1.zip › Figure 1_Source data 1.pdf]

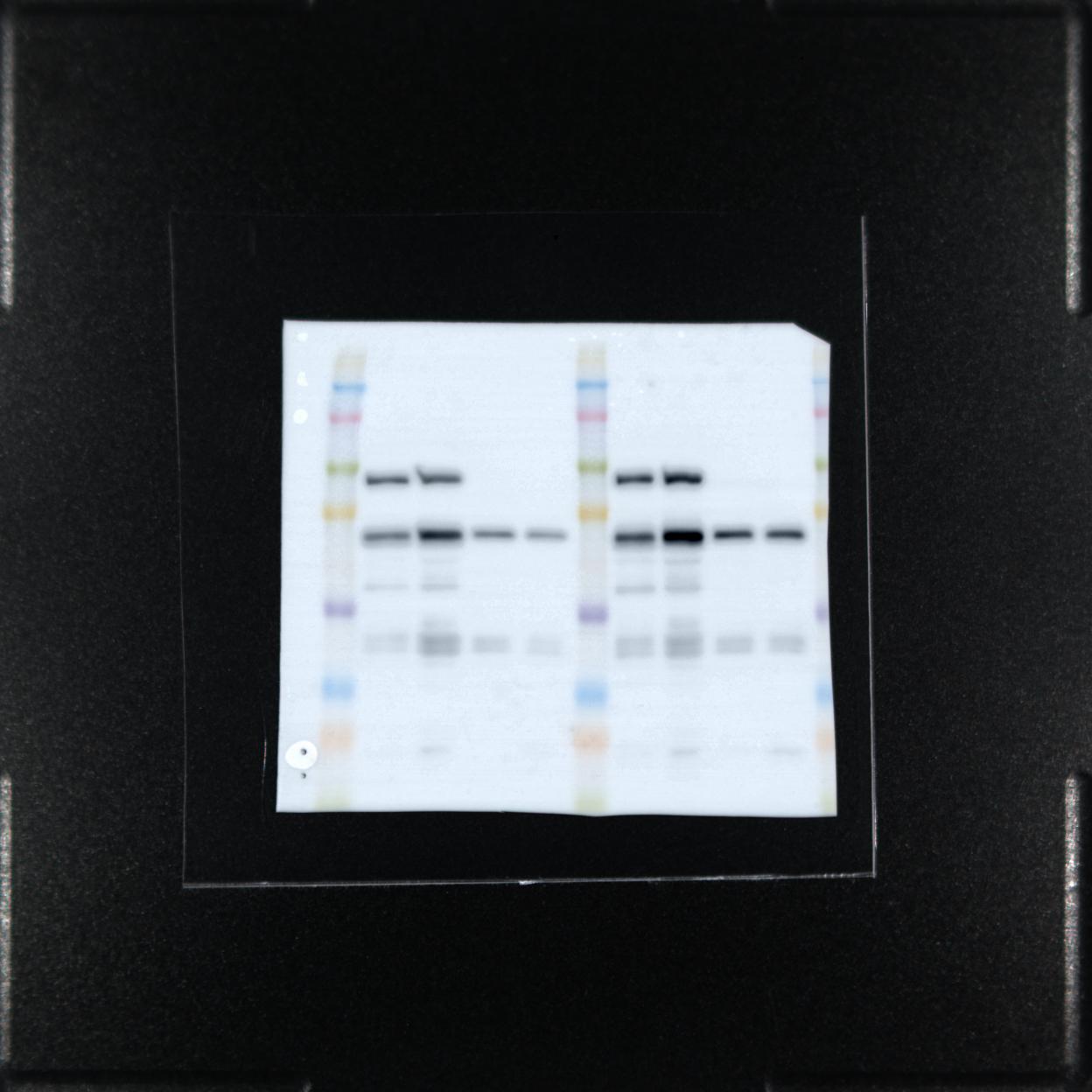

Supplement: Figure 1—source data 2. [file elife-104432-fig1-data2.zip › Figure 1_Source data 2/Fig1a_RAF.jpg]

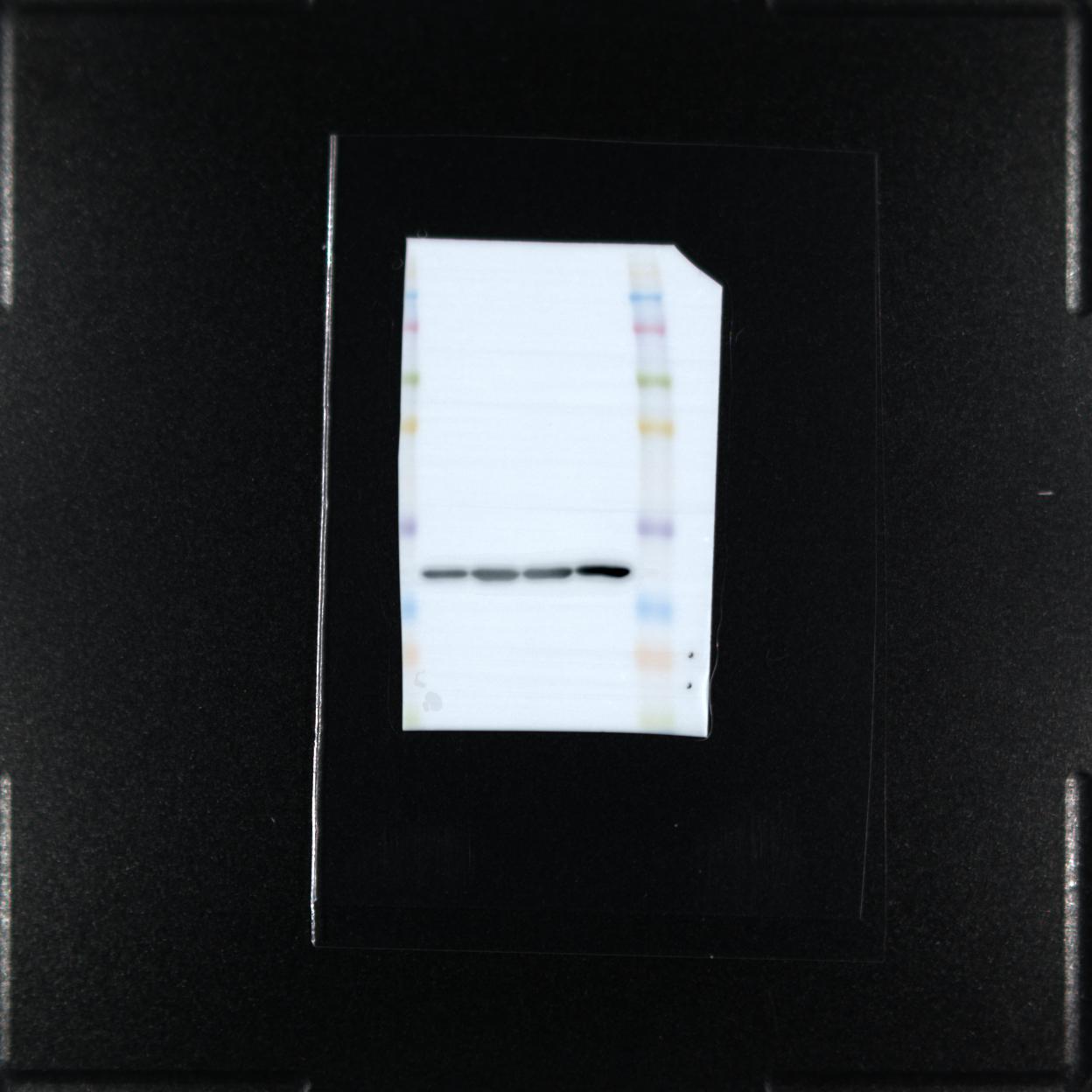

Supplement: Figure 1—source data 2. [file elife-104432-fig1-data2.zip › Figure 1_Source data 2/Fig1a_actin_2 .jpg]

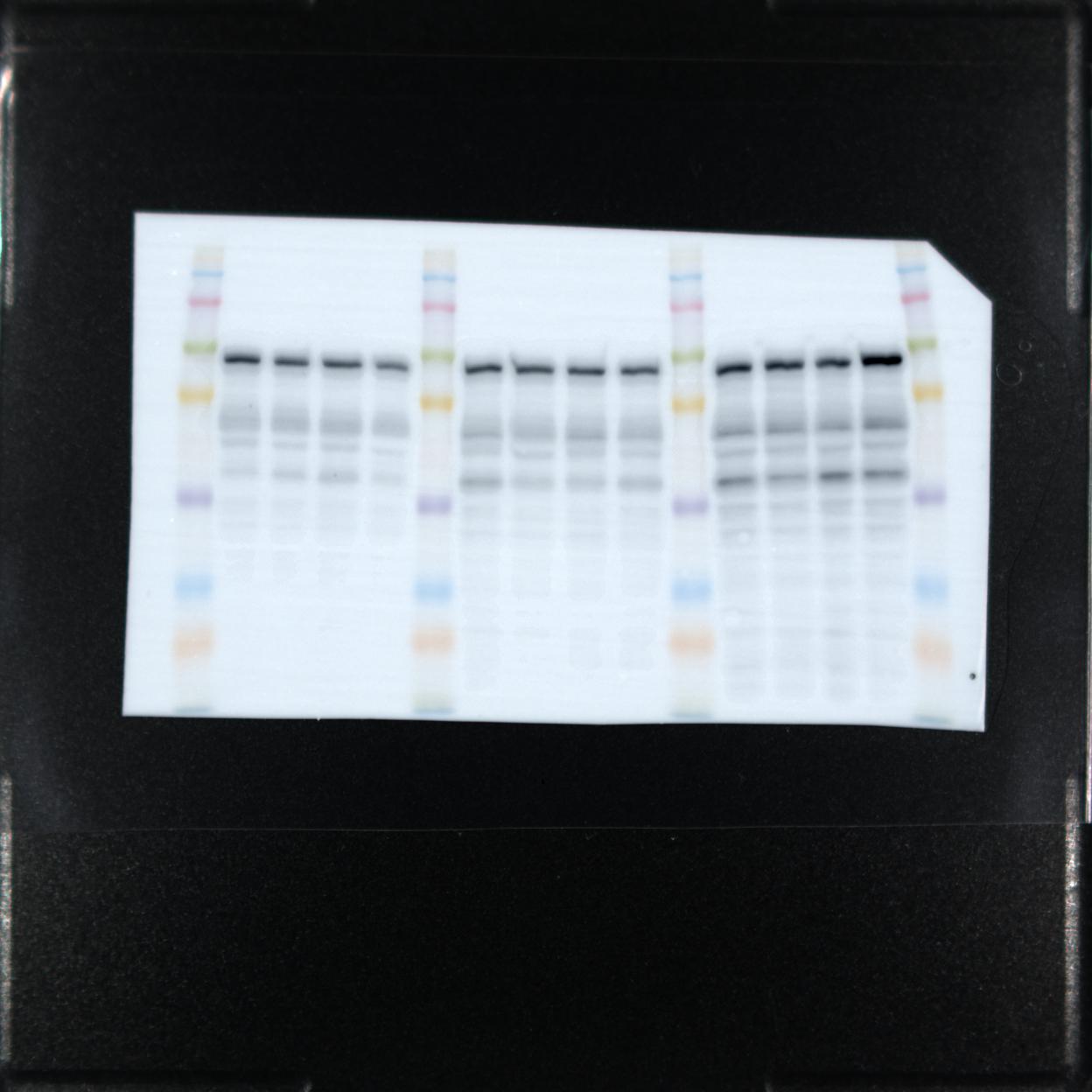

Supplement: Figure 1—source data 2. [file elife-104432-fig1-data2.zip › Figure 1_Source data 2/Fig1b_RAF.jpg]

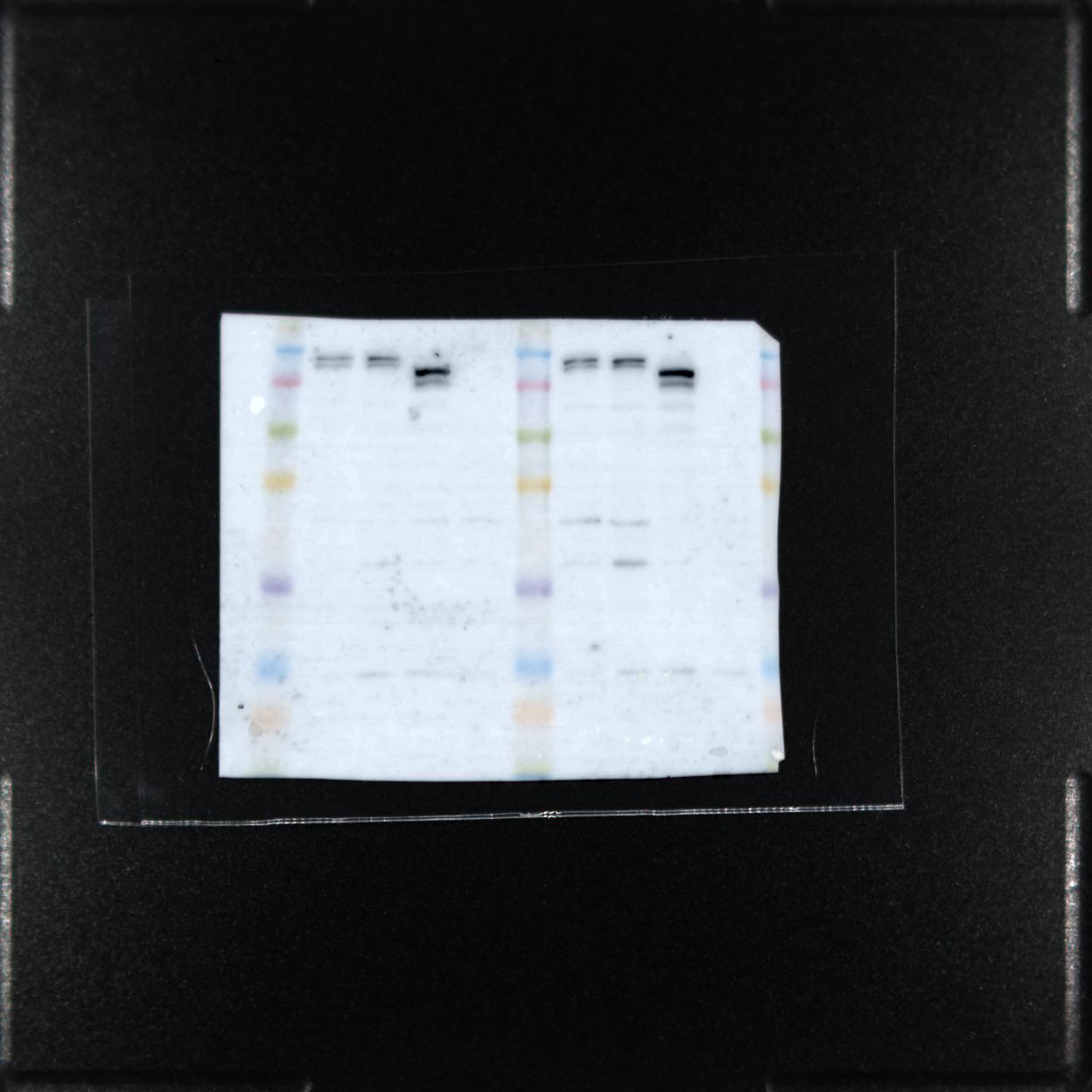

Supplement: Figure 1—source data 2. [file elife-104432-fig1-data2.zip › Figure 1_Source data 2/Fig1a_SOS.jpg]

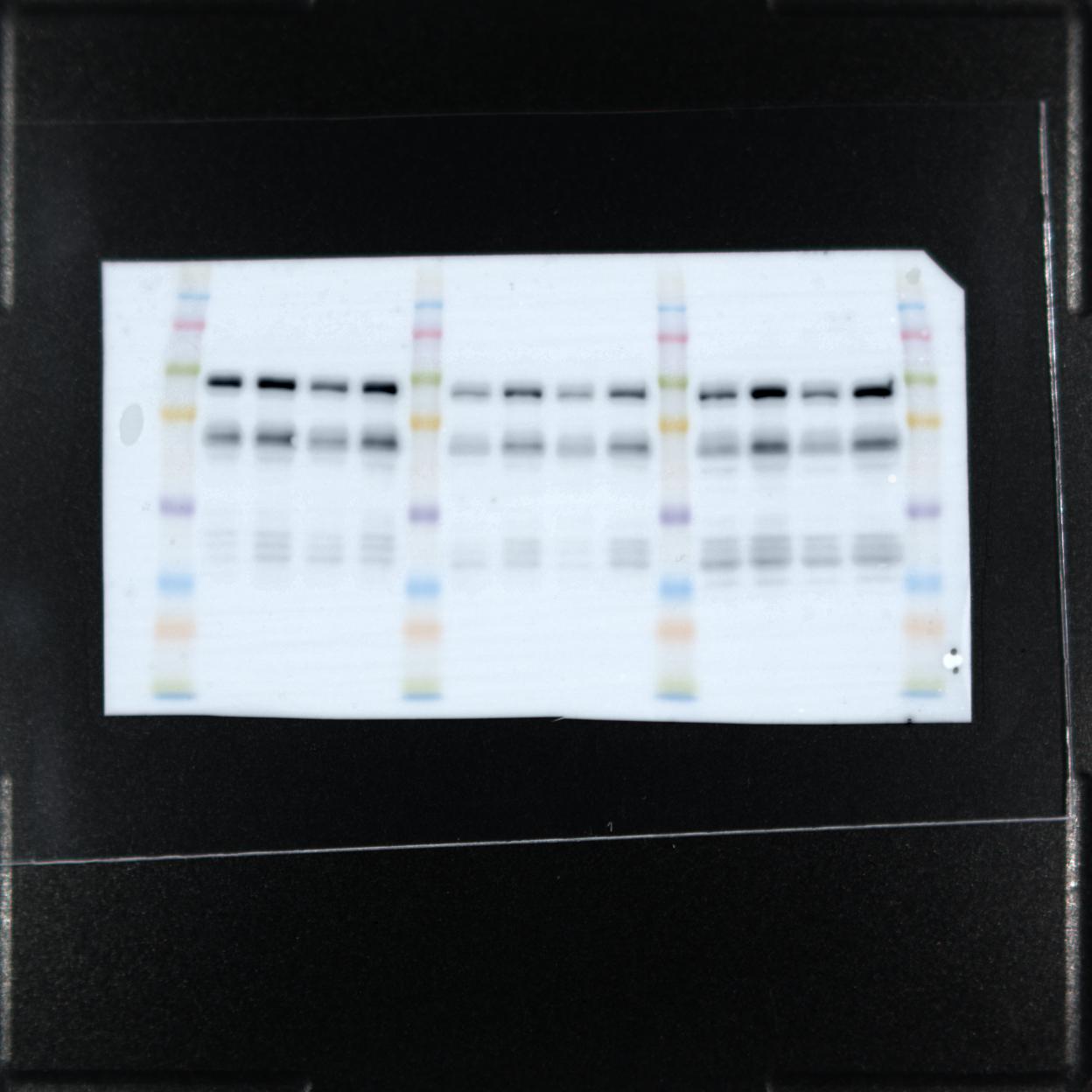

Supplement: Figure 1—source data 2. [file elife-104432-fig1-data2.zip › Figure 1_Source data 2/Fig1b_pS338.jpg]

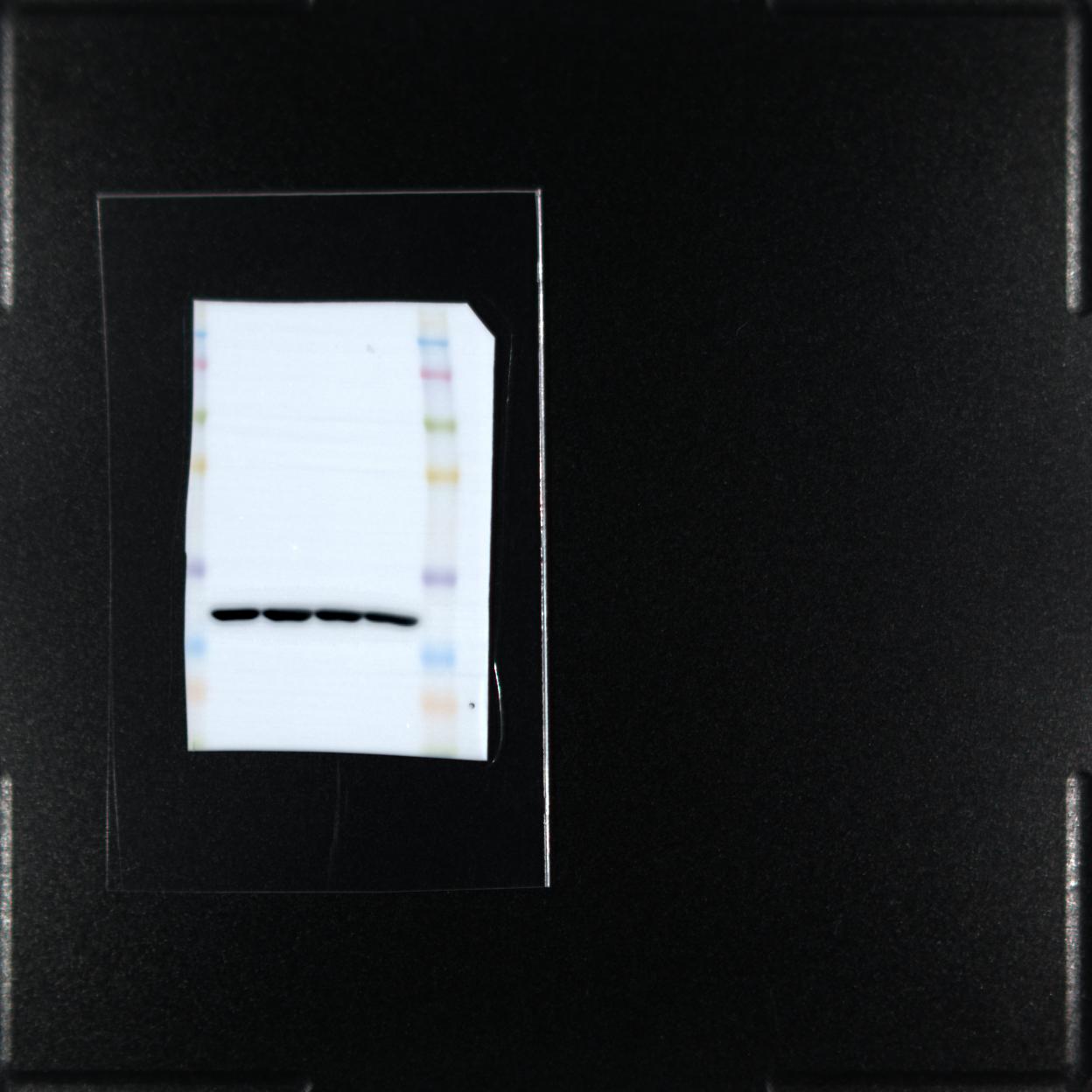

Supplement: Figure 1—source data 2. [file elife-104432-fig1-data2.zip › Figure 1_Source data 2/Fig1a_actin_1 .jpg]

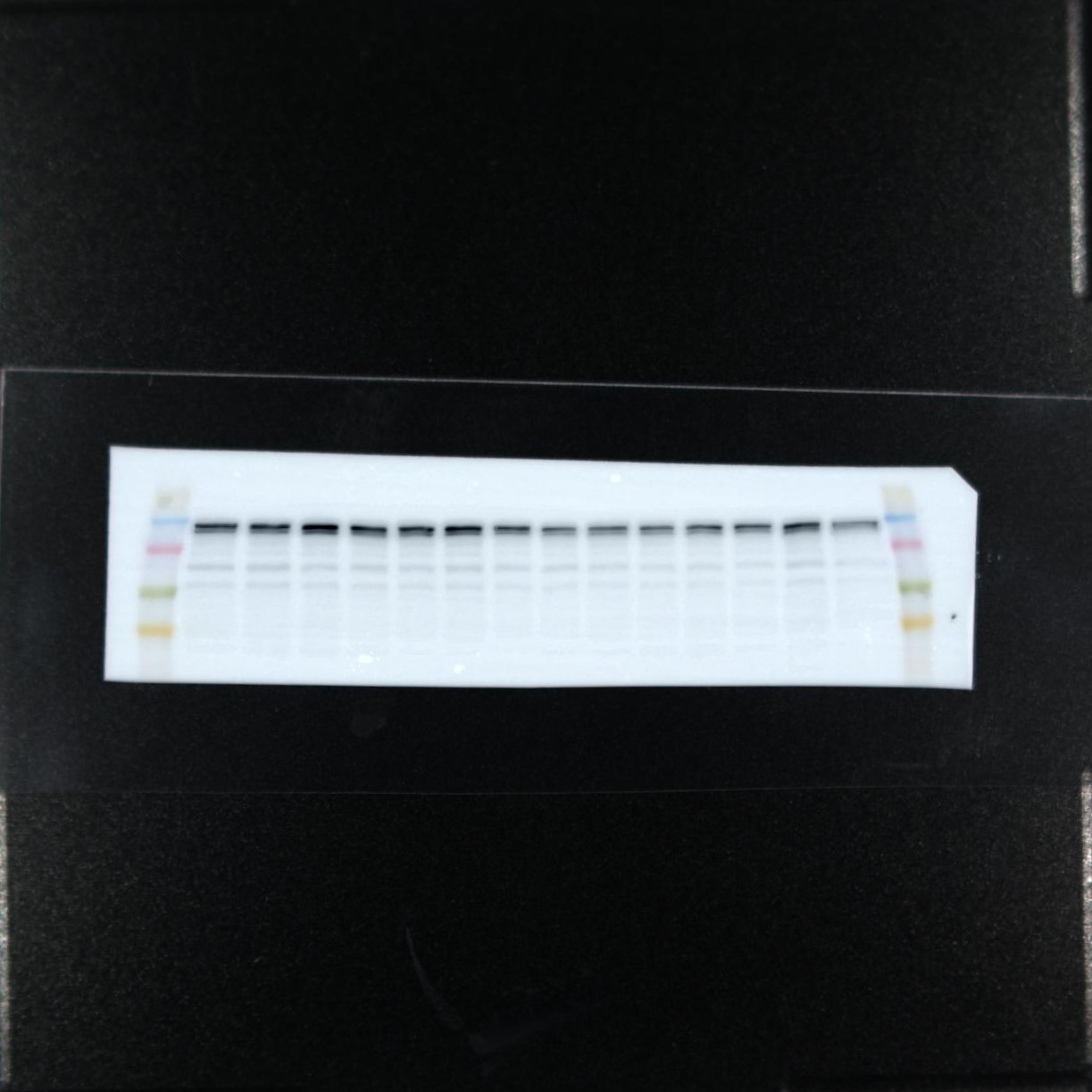

Supplement: Figure 5—source data 2. [file elife-104432-fig5-data2.zip › Figure 5_Source data 2/Fig5a_SOS_1.jpg]

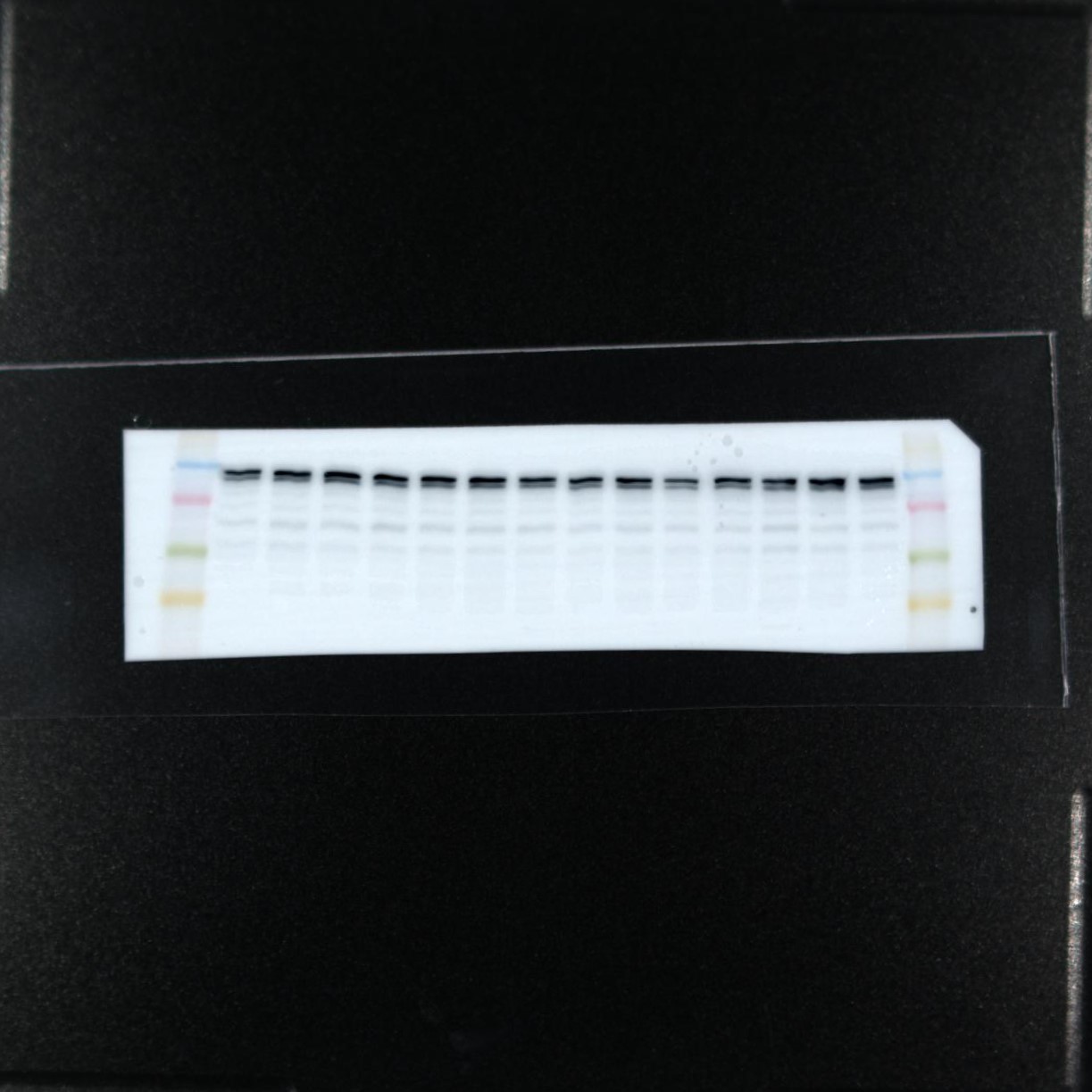

Supplement: Figure 5—source data 2. [file elife-104432-fig5-data2.zip › Figure 5_Source data 2/Fig5a_SOS_3.jpg]

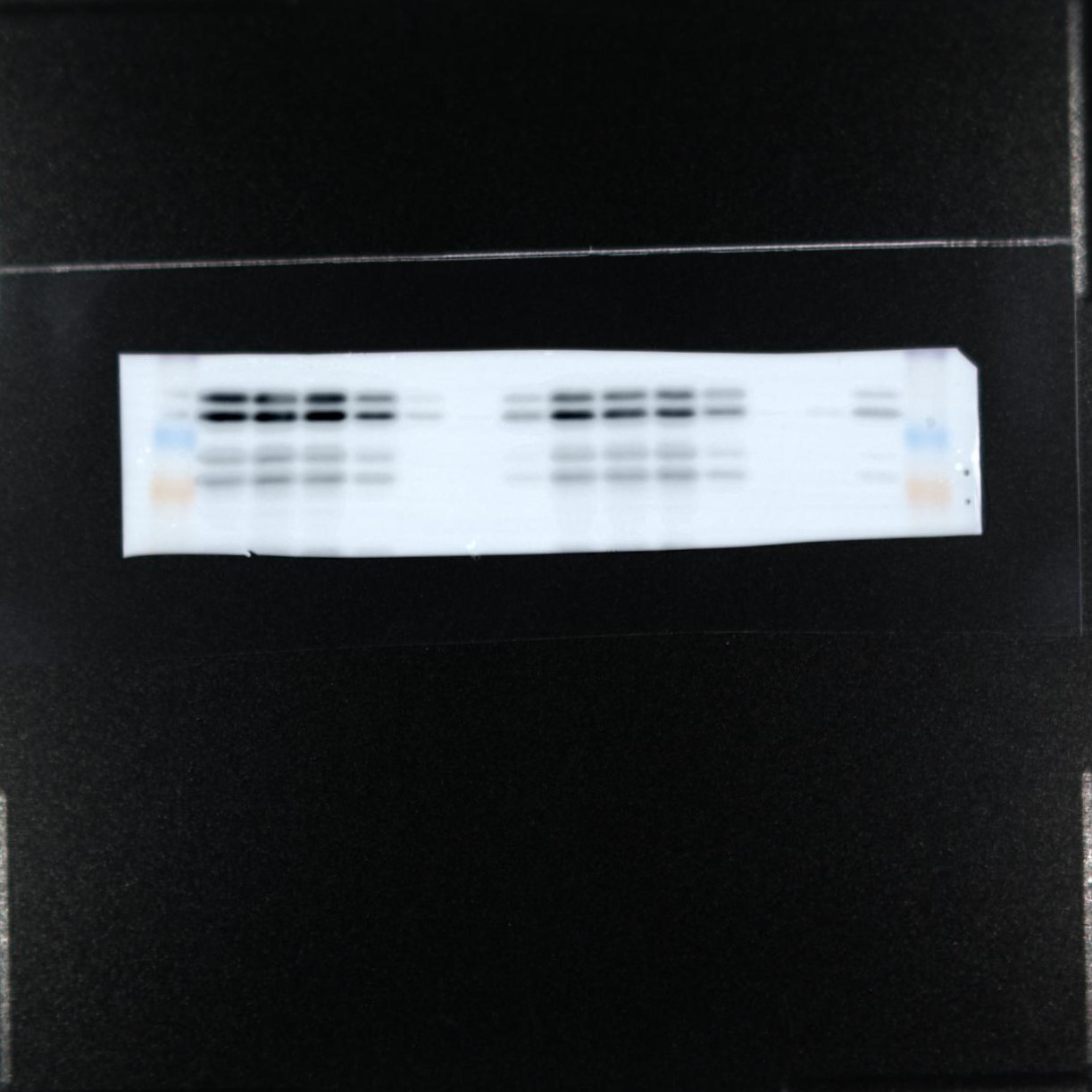

Supplement: Figure 5—source data 2. [file elife-104432-fig5-data2.zip › Figure 5_Source data 2/Fig5a_ppERK_4.jpg]

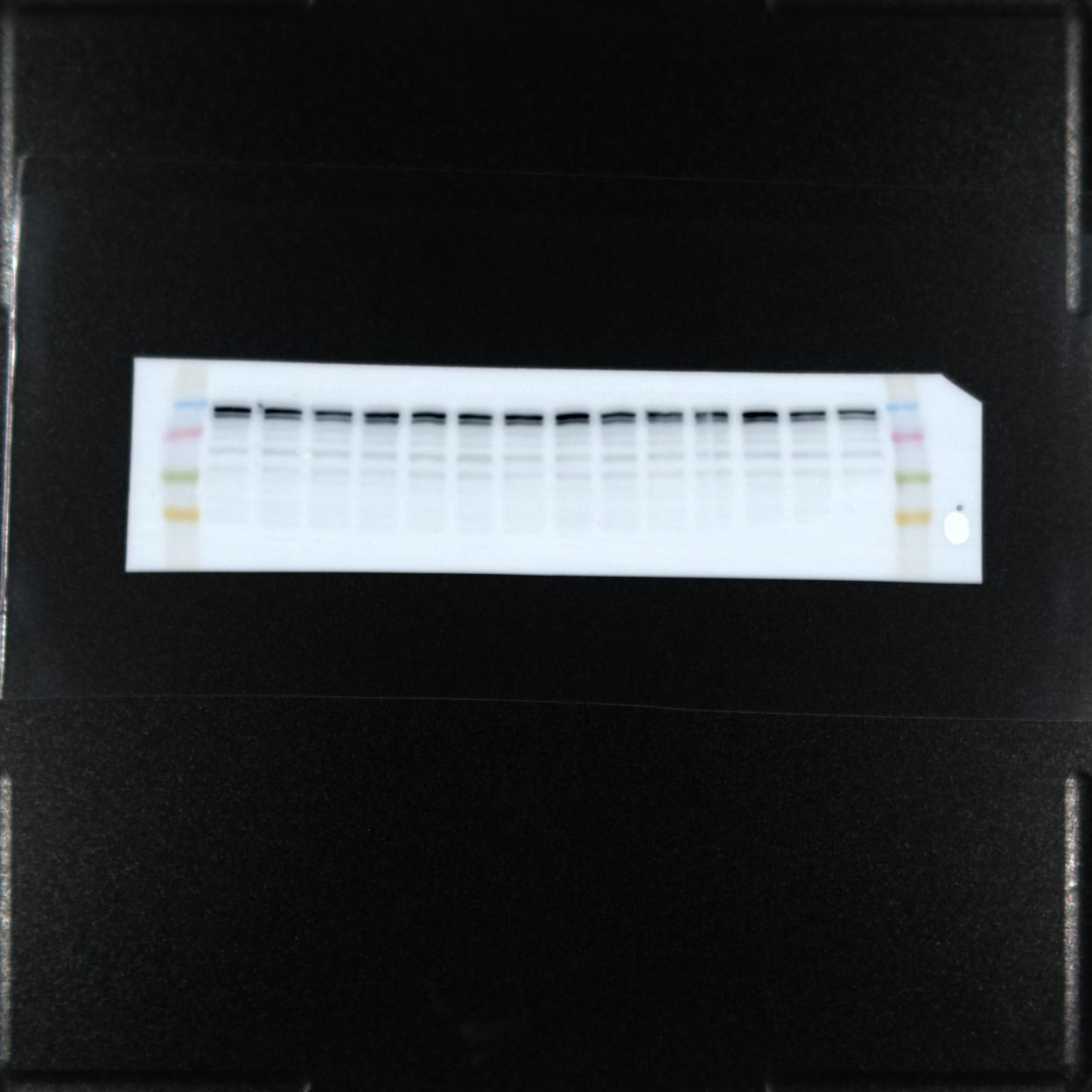

Supplement: Figure 5—source data 2. [file elife-104432-fig5-data2.zip › Figure 5_Source data 2/Fig5a_SOS_2.jpg]

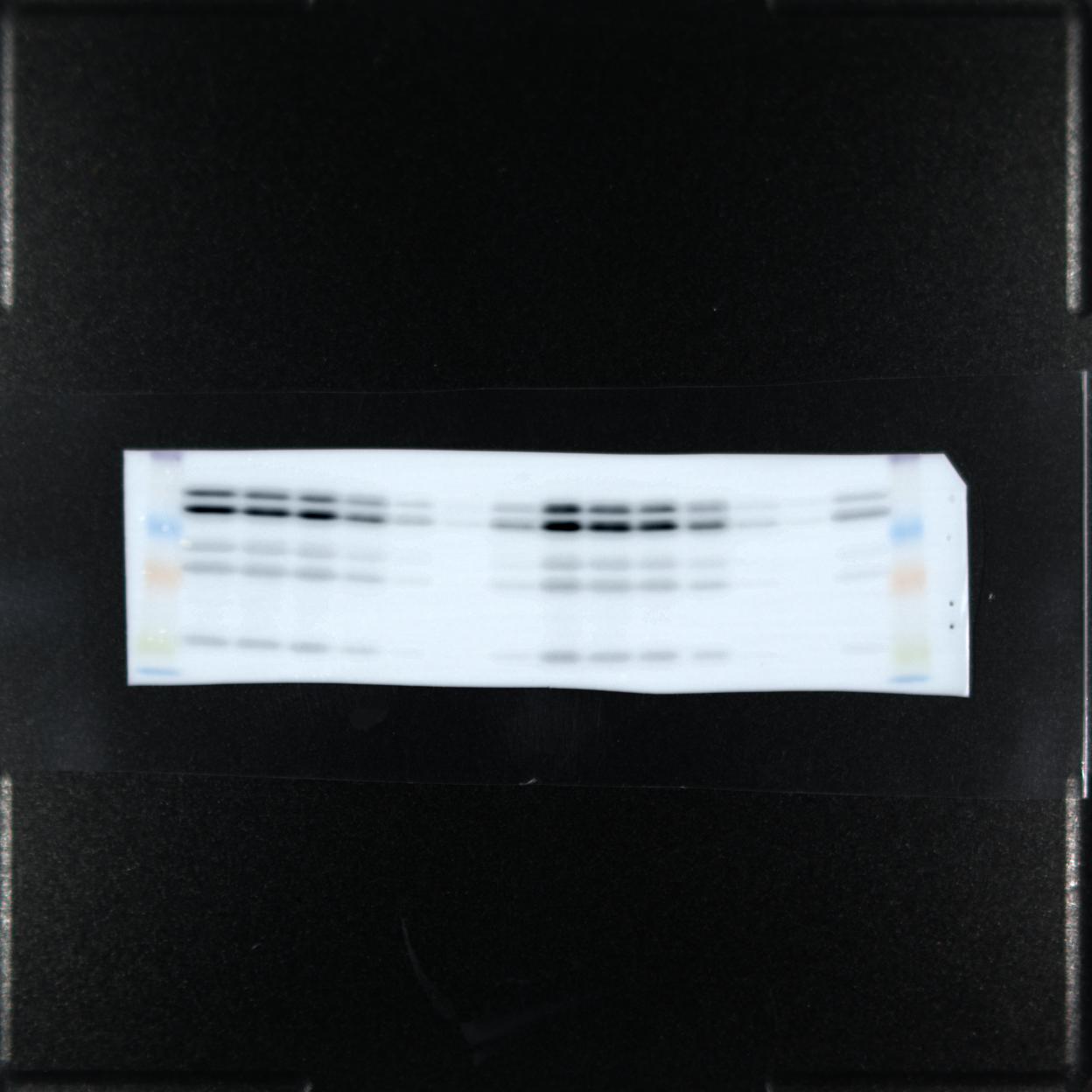

Supplement: Figure 5—source data 2. [file elife-104432-fig5-data2.zip › Figure 5_Source data 2/Fig5a_ppERK_1.jpg]

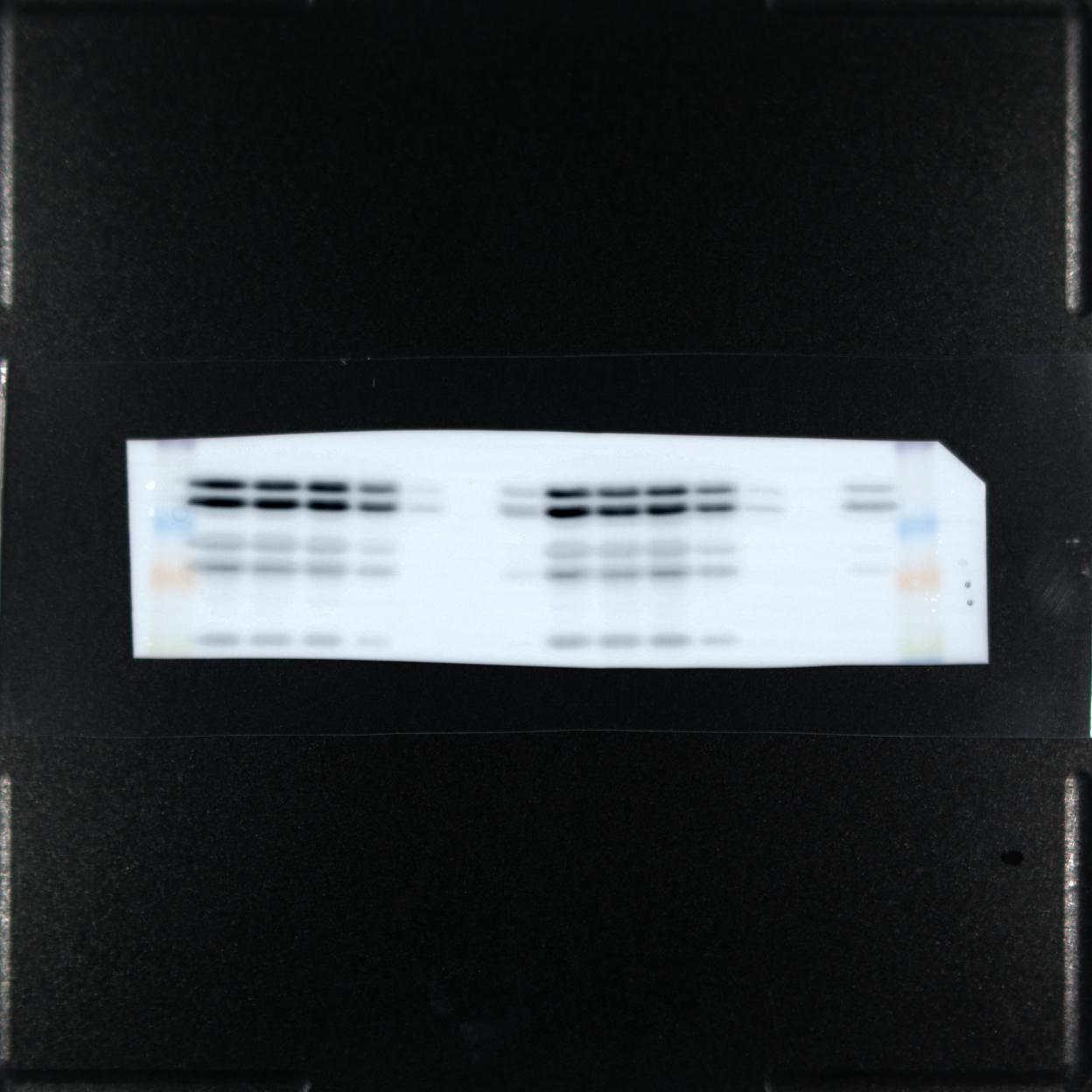

Supplement: Figure 5—source data 2. [file elife-104432-fig5-data2.zip › Figure 5_Source data 2/Fig5a_ppERK_3.jpg]

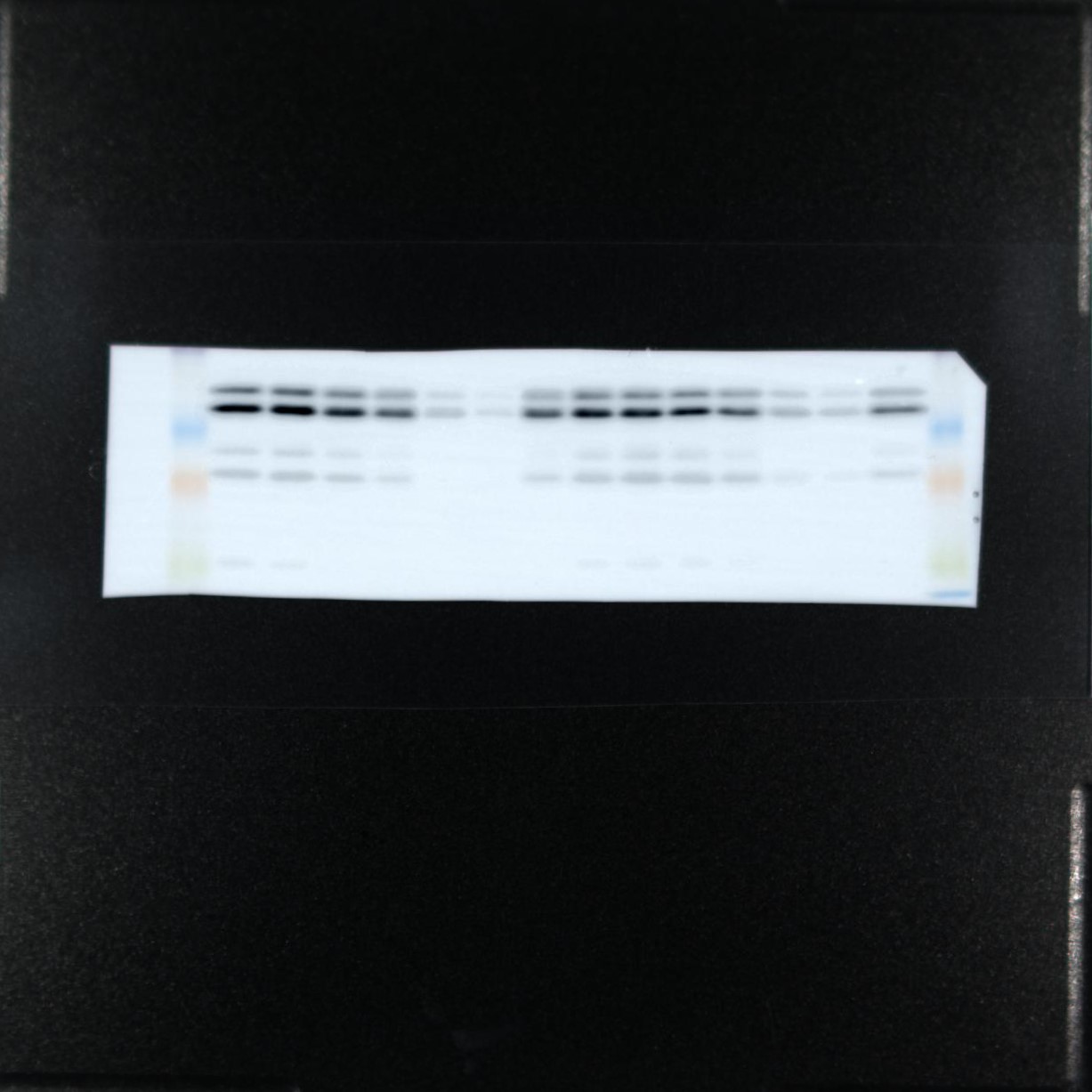

Supplement: Figure 5—source data 2. [file elife-104432-fig5-data2.zip › Figure 5_Source data 2/Fig5a_ppERK_2.jpg]

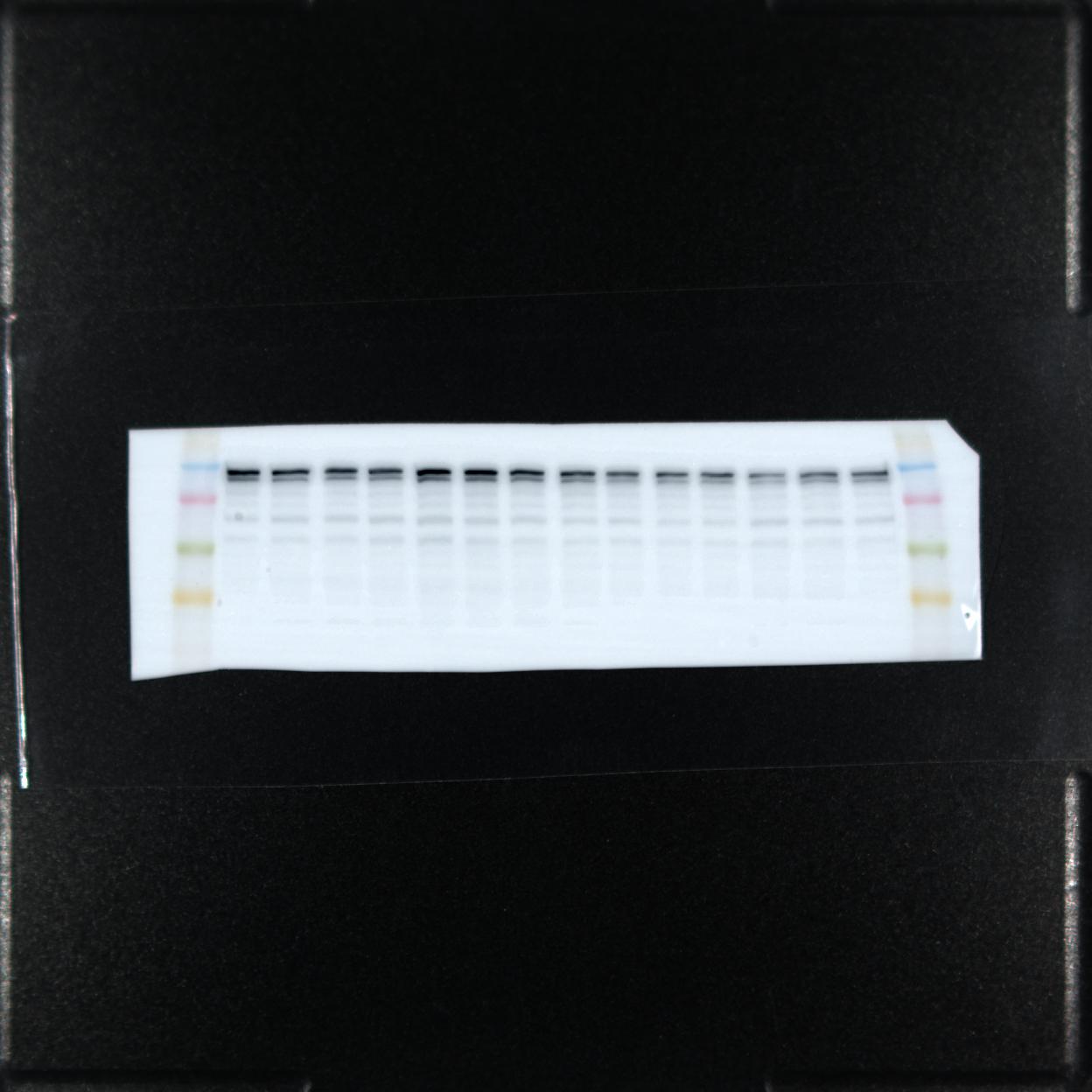

Supplement: Figure 5—source data 2. [file elife-104432-fig5-data2.zip › Figure 5_Source data 2/Fig5a_SOS_4.jpg]

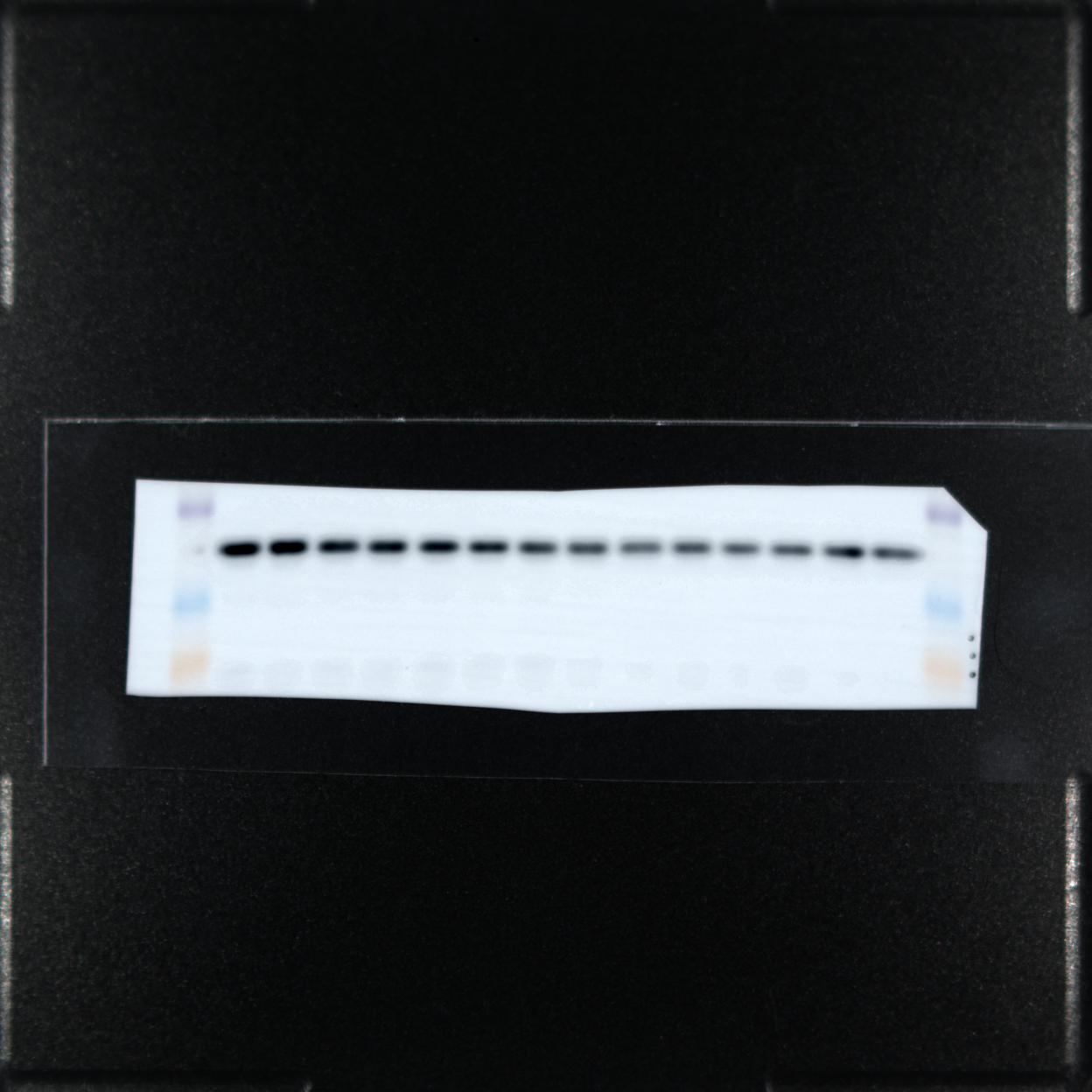

Supplement: Figure 5—source data 2. [file elife-104432-fig5-data2.zip › Figure 5_Source data 2/Fig5a_MEK_4.jpg]

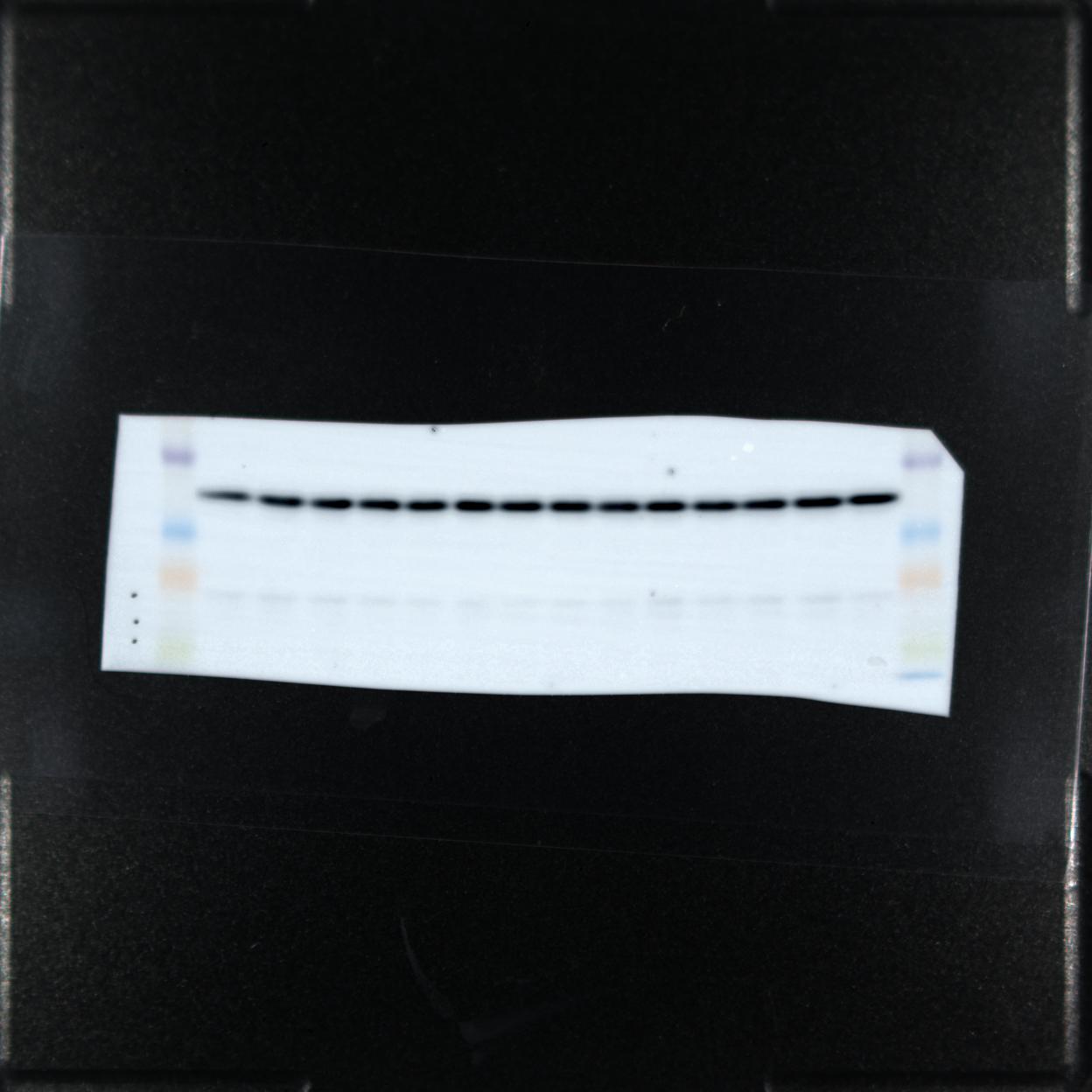

Supplement: Figure 5—source data 2. [file elife-104432-fig5-data2.zip › Figure 5_Source data 2/Fig5a_MEK_1.jpg]

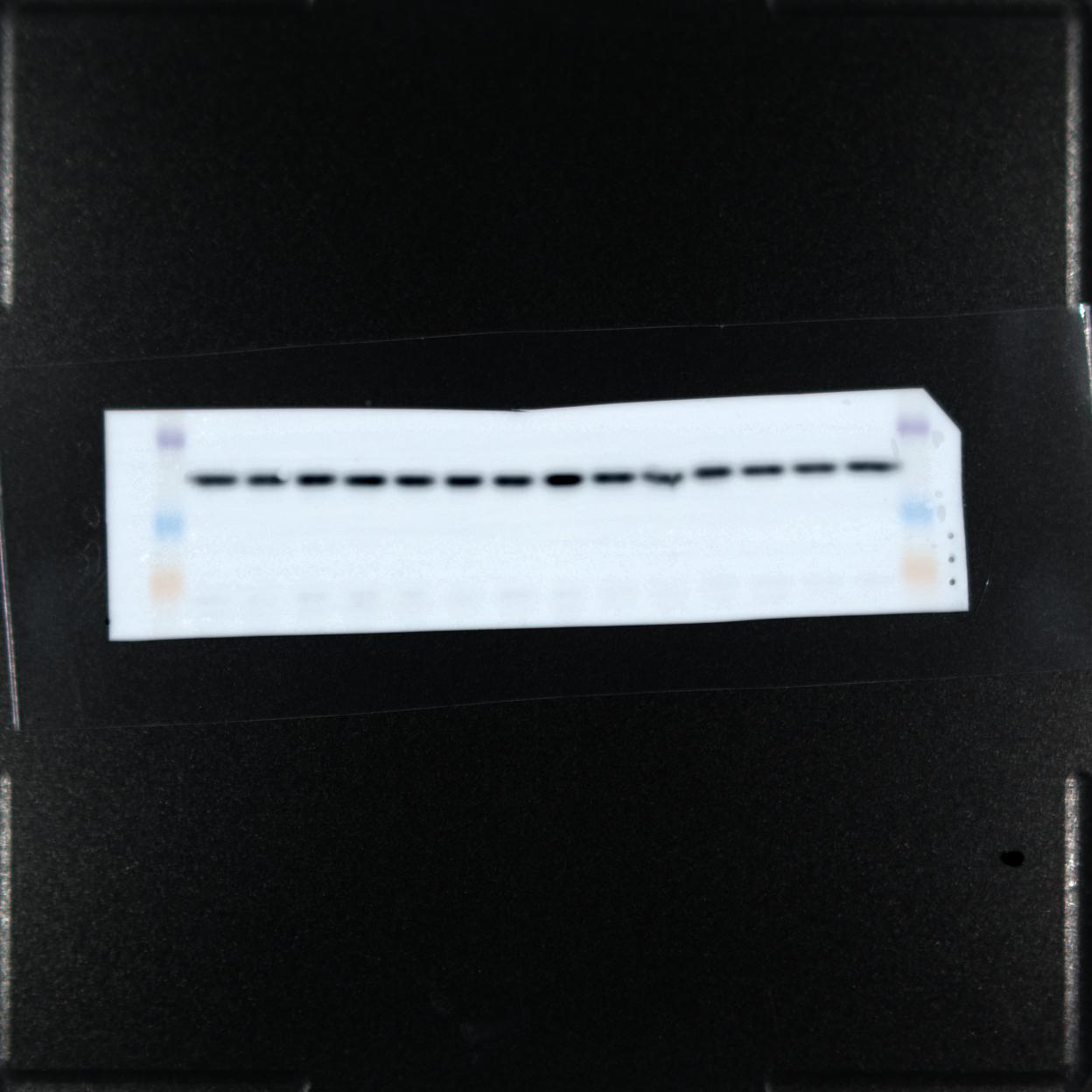

Supplement: Figure 5—source data 2. [file elife-104432-fig5-data2.zip › Figure 5_Source data 2/Fig5a_MEK_3.jpg]

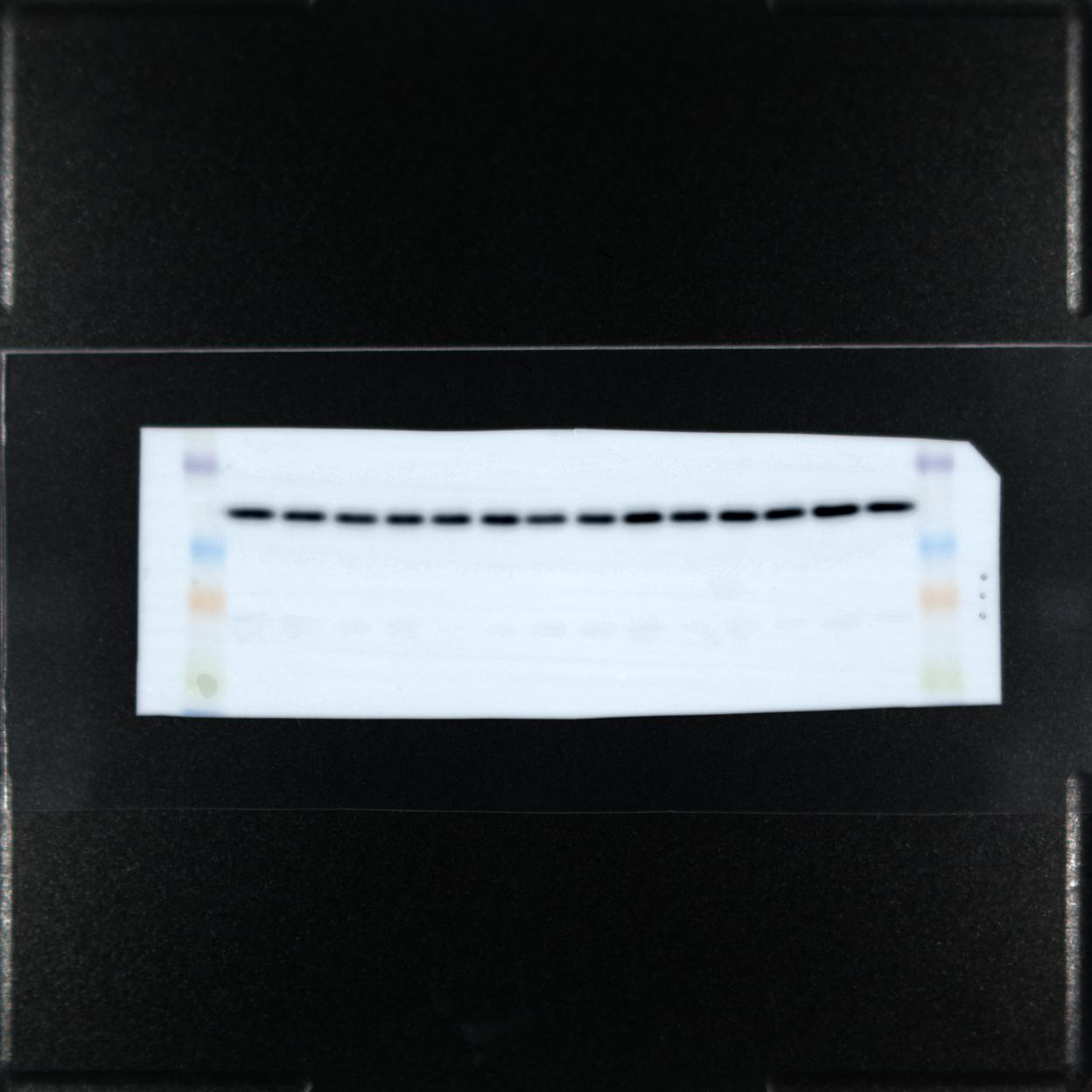

Supplement: Figure 5—source data 2. [file elife-104432-fig5-data2.zip › Figure 5_Source data 2/Fig5a_MEK_2.jpg]

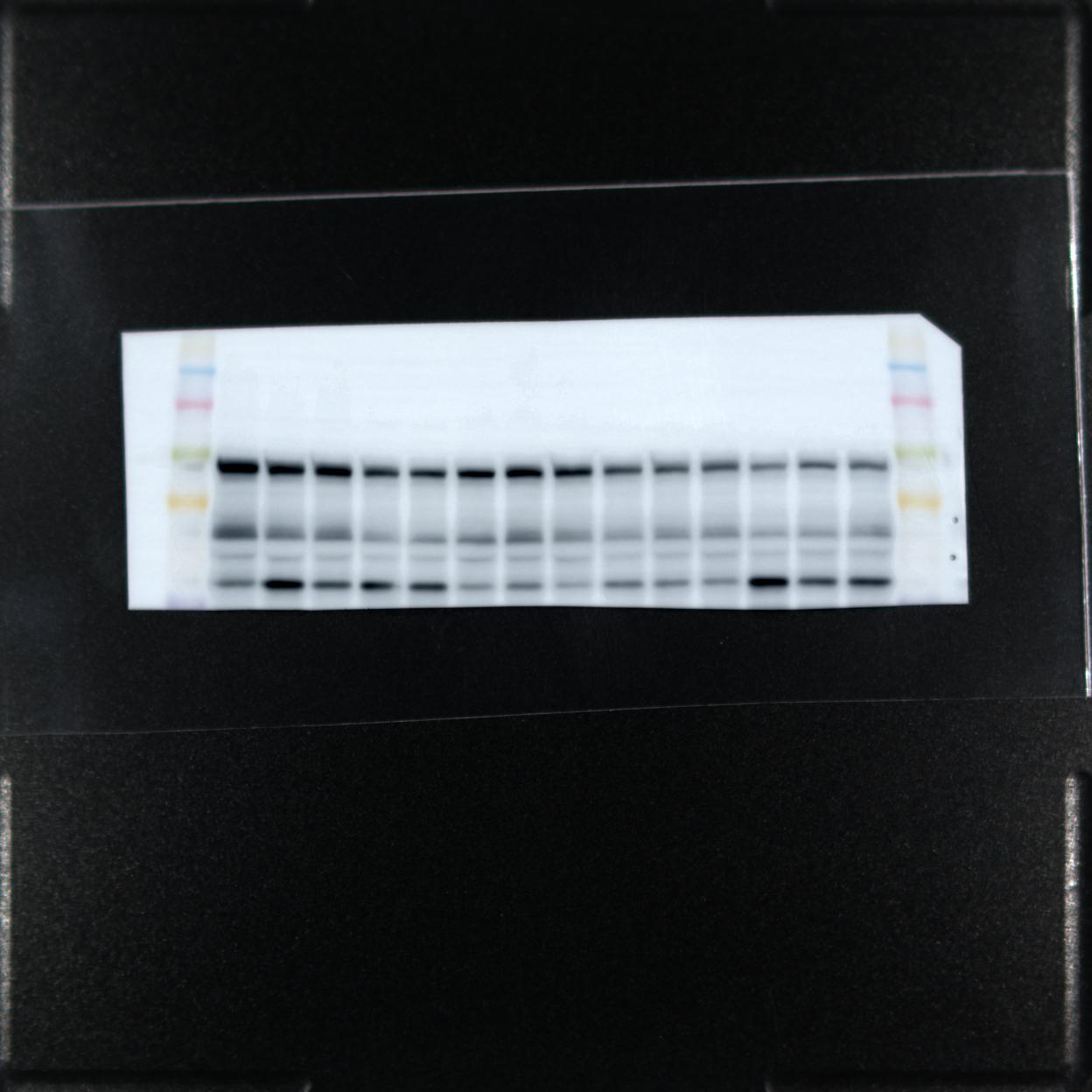

Supplement: Figure 5—source data 2. [file elife-104432-fig5-data2.zip › Figure 5_Source data 2/Fig5a_RAF_4.jpg]

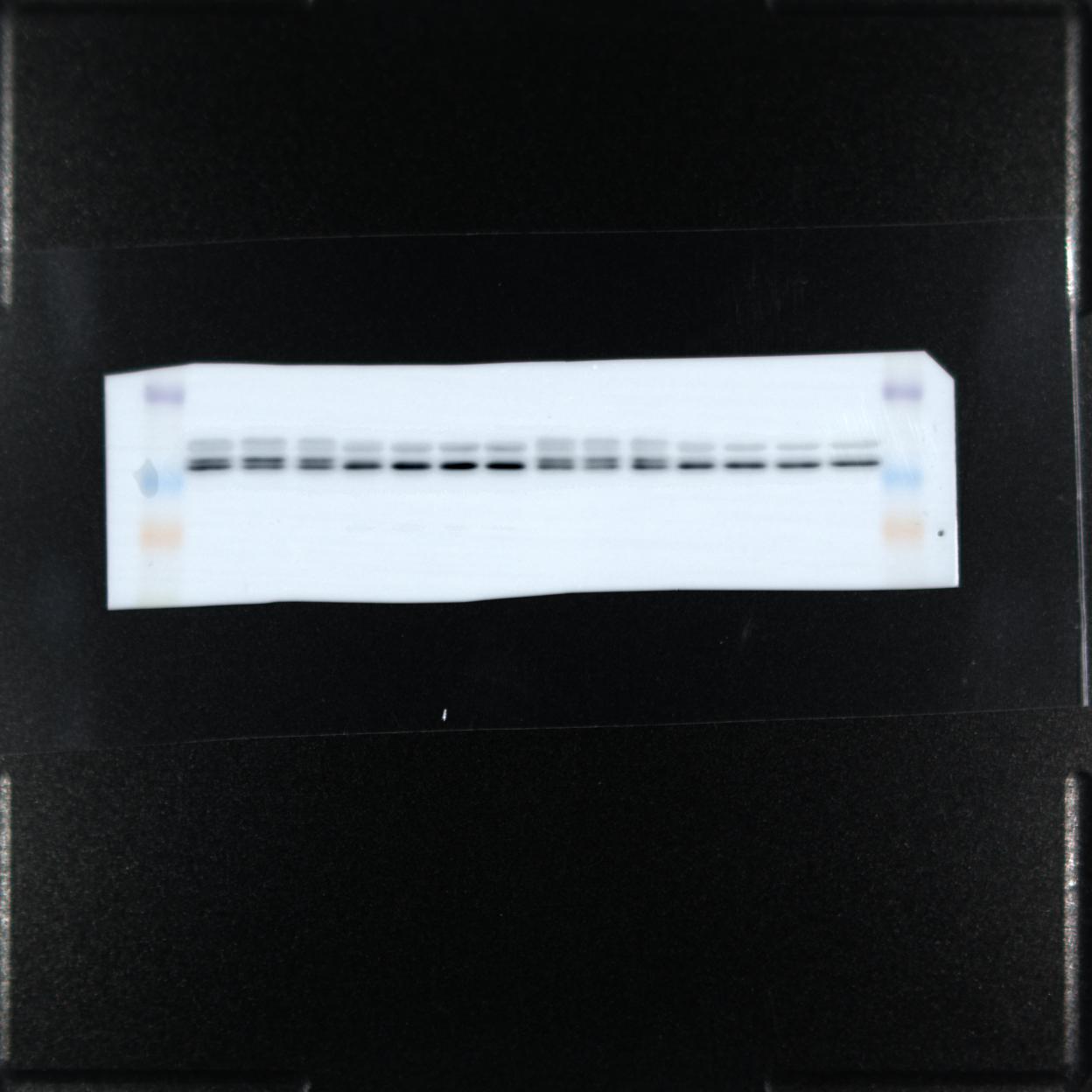

Supplement: Figure 5—source data 2. [file elife-104432-fig5-data2.zip › Figure 5_Source data 2/Fig5a_ERK_4.jpg]

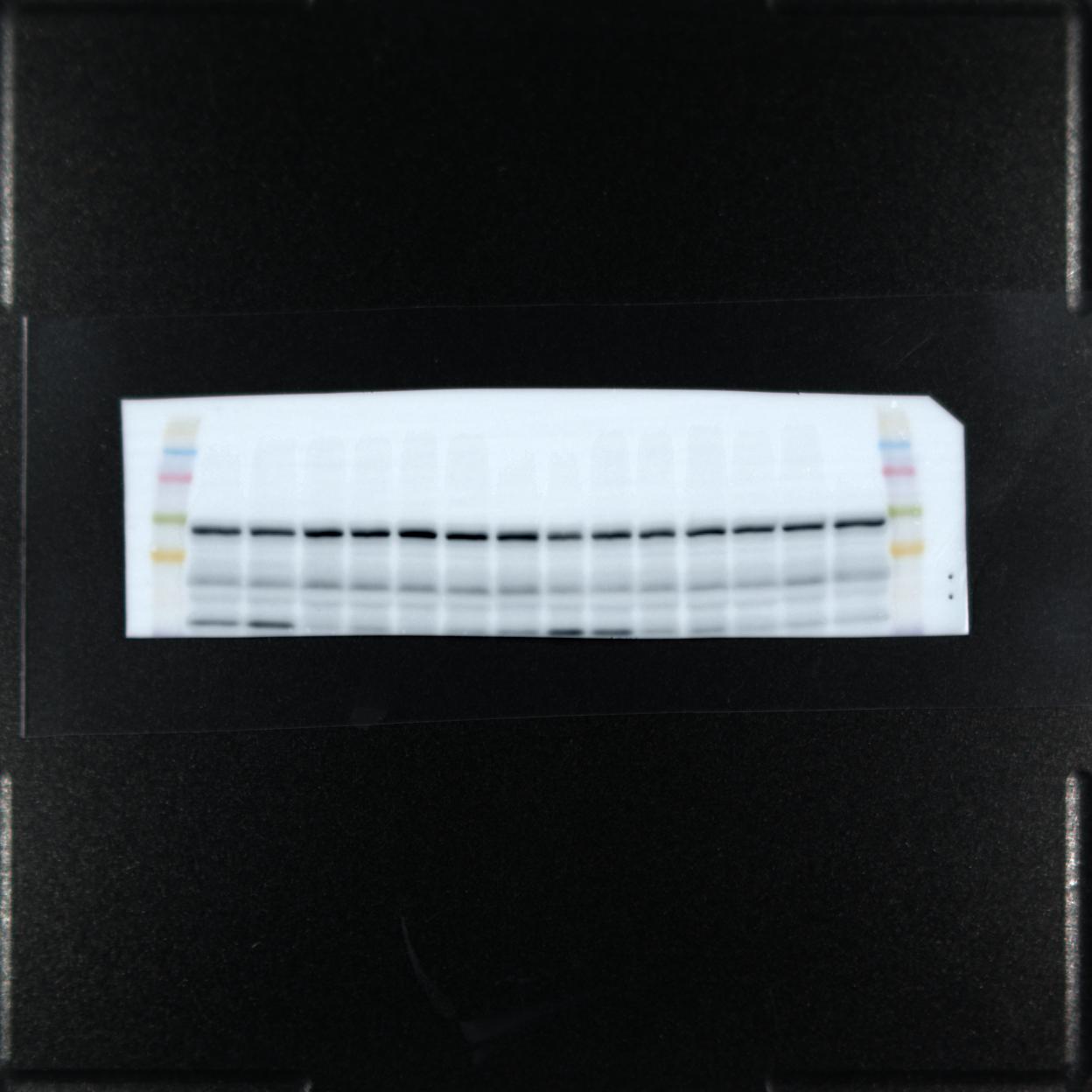

Supplement: Figure 5—source data 2. [file elife-104432-fig5-data2.zip › Figure 5_Source data 2/Fig5a_RAF_1.jpg]

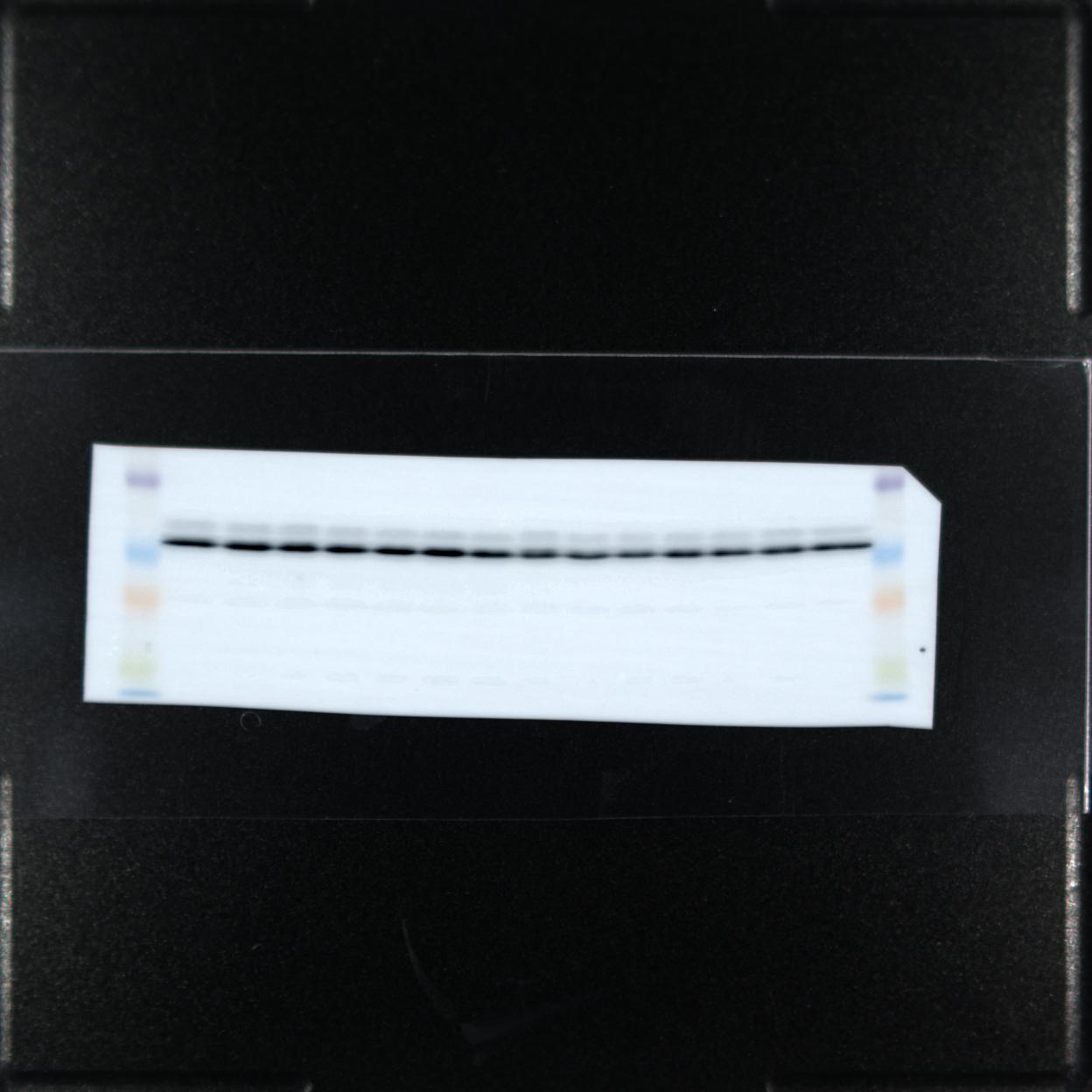

Supplement: Figure 5—source data 2. [file elife-104432-fig5-data2.zip › Figure 5_Source data 2/Fig5a_ERK_1.jpg]

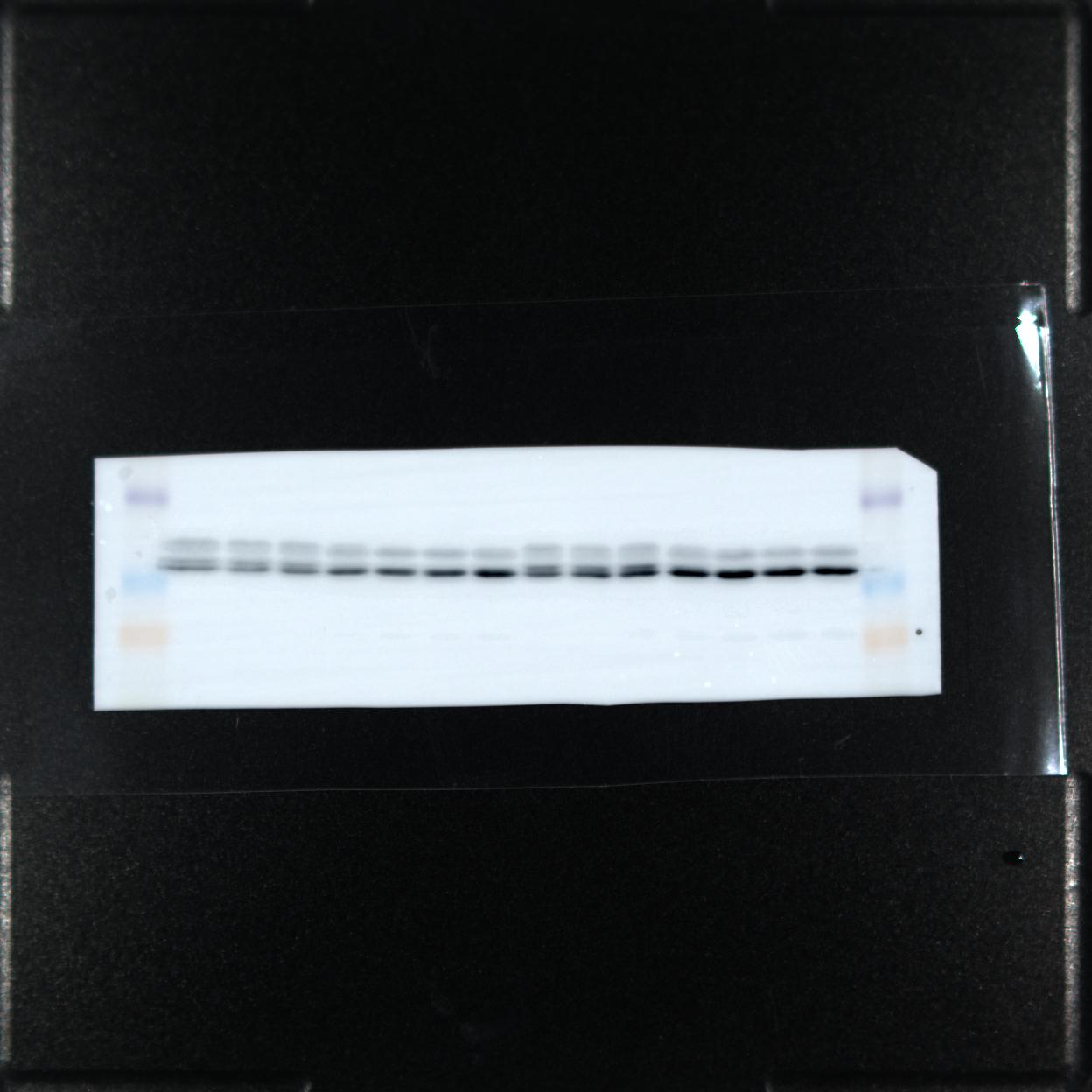

Supplement: Figure 5—source data 2. [file elife-104432-fig5-data2.zip › Figure 5_Source data 2/Fig5a_ERK_3.jpg]

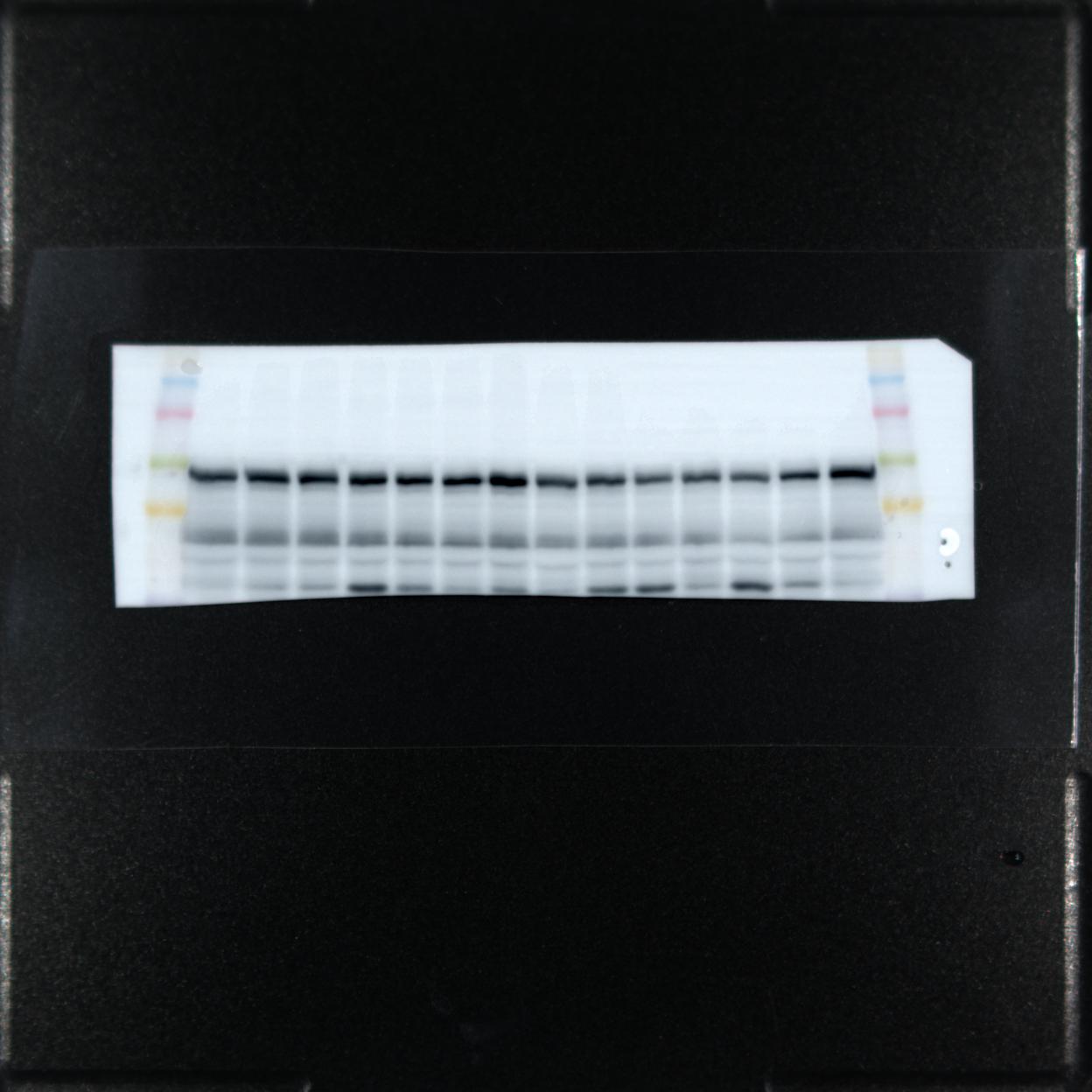

Supplement: Figure 5—source data 2. [file elife-104432-fig5-data2.zip › Figure 5_Source data 2/Fig5a_RAF_3.jpg]

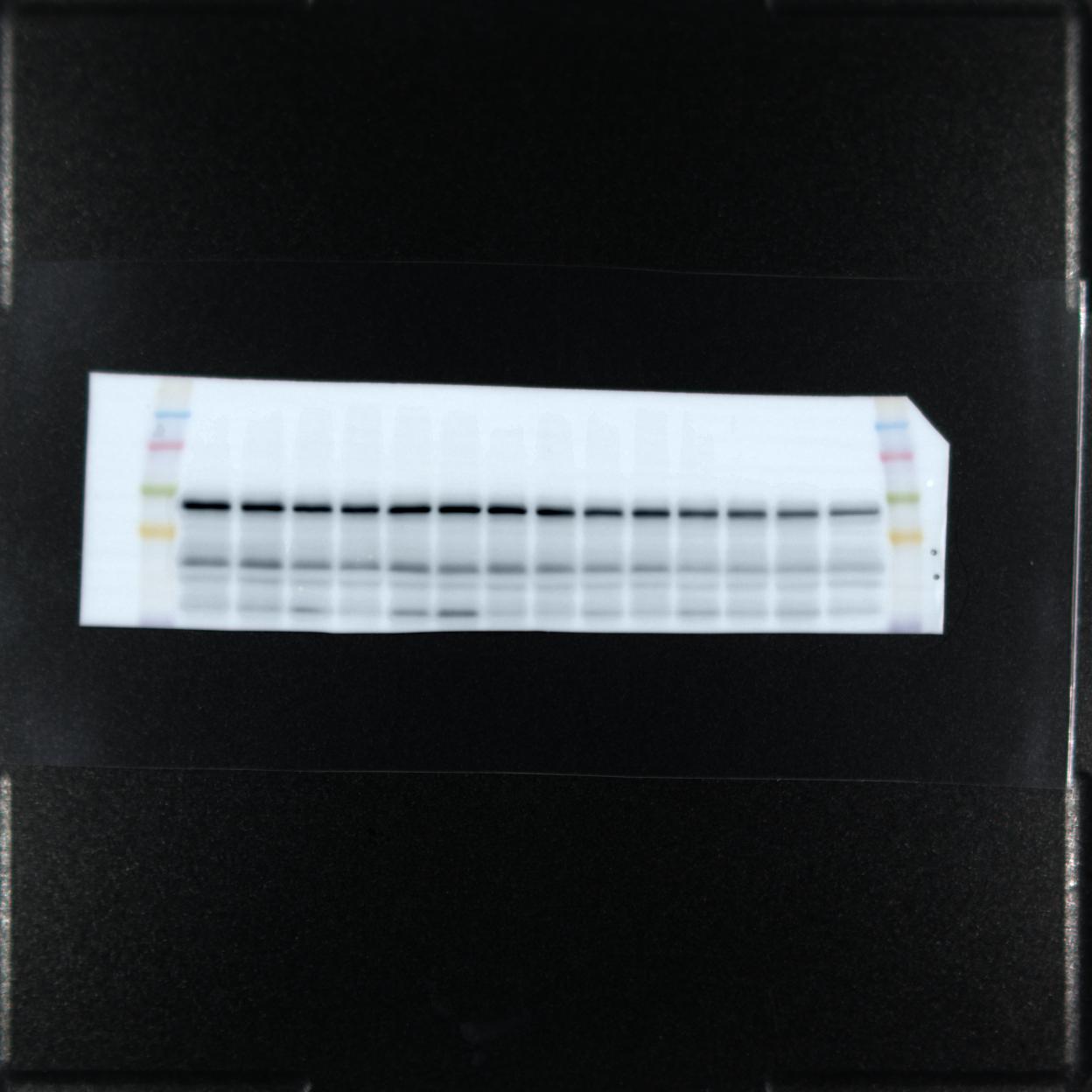

Supplement: Figure 5—source data 2. [file elife-104432-fig5-data2.zip › Figure 5_Source data 2/Fig5a_RAF_2.jpg]

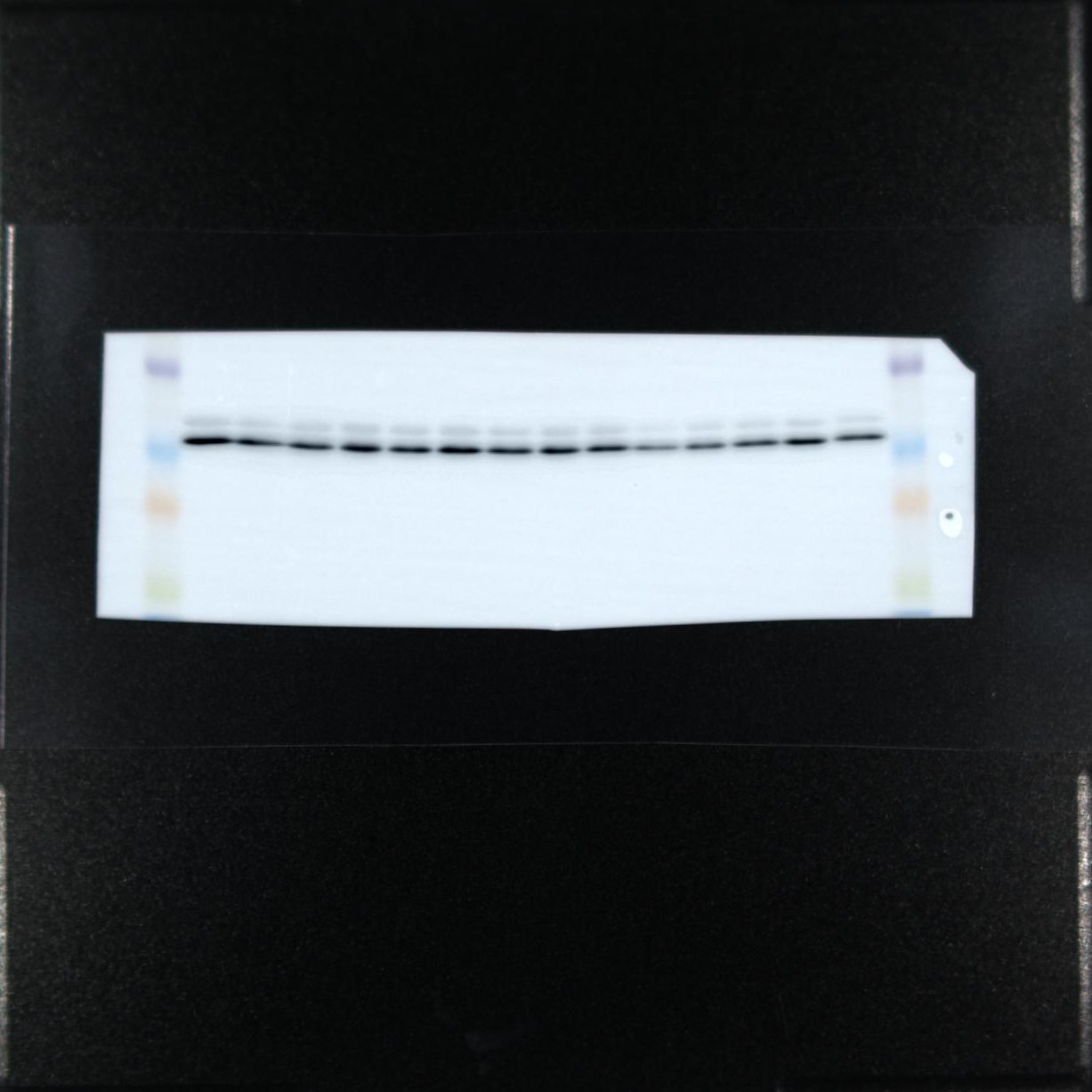

Supplement: Figure 5—source data 2. [file elife-104432-fig5-data2.zip › Figure 5_Source data 2/Fig5a_ERK_2.jpg]

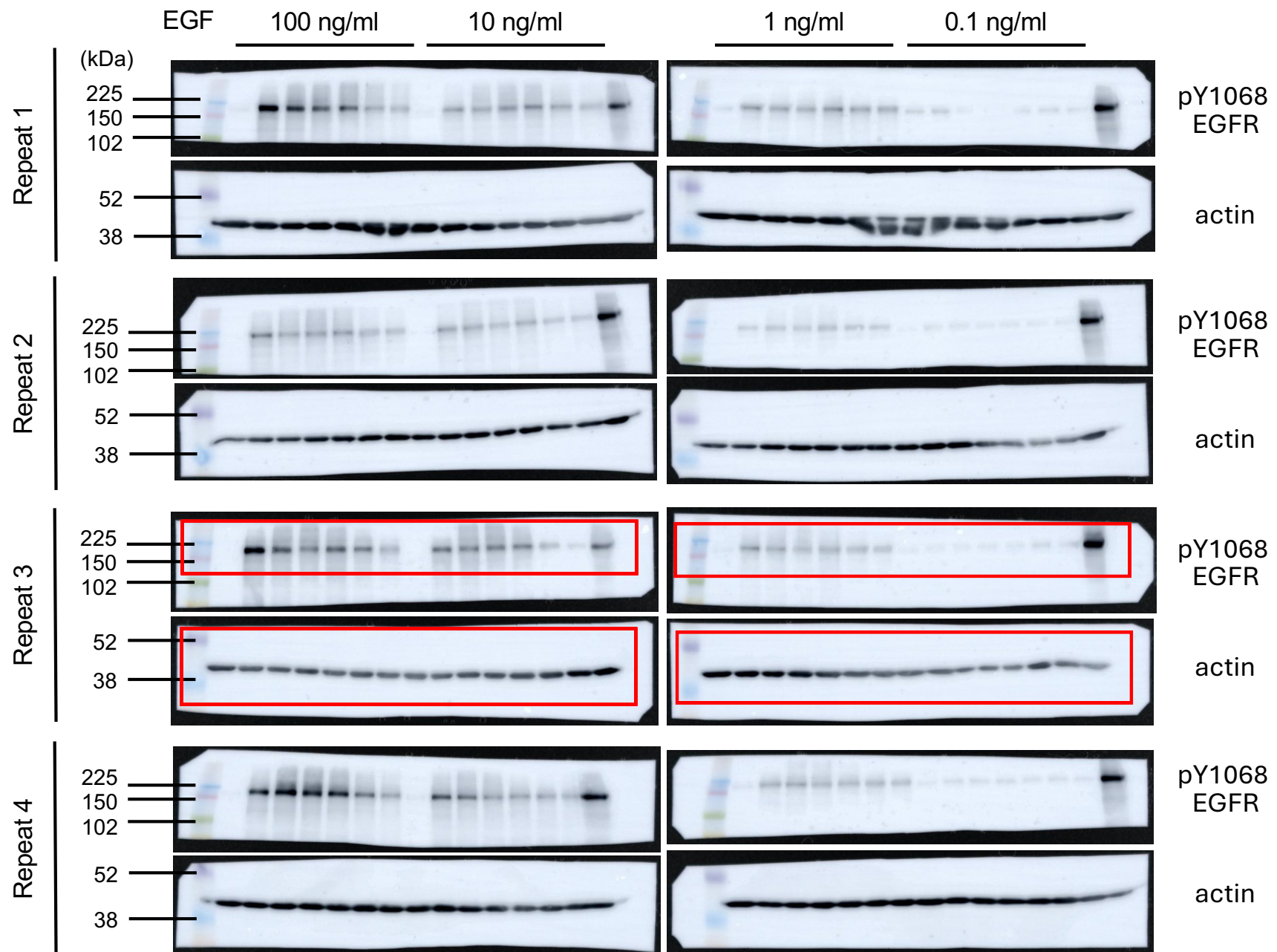

Appendix Figure 10\_Source data 1

Supplement: Appendix 1—figure 10—source data 1. [file elife-104432-app1-fig10-data1.zip › Appendix Figure 10_Source data 1.pdf]

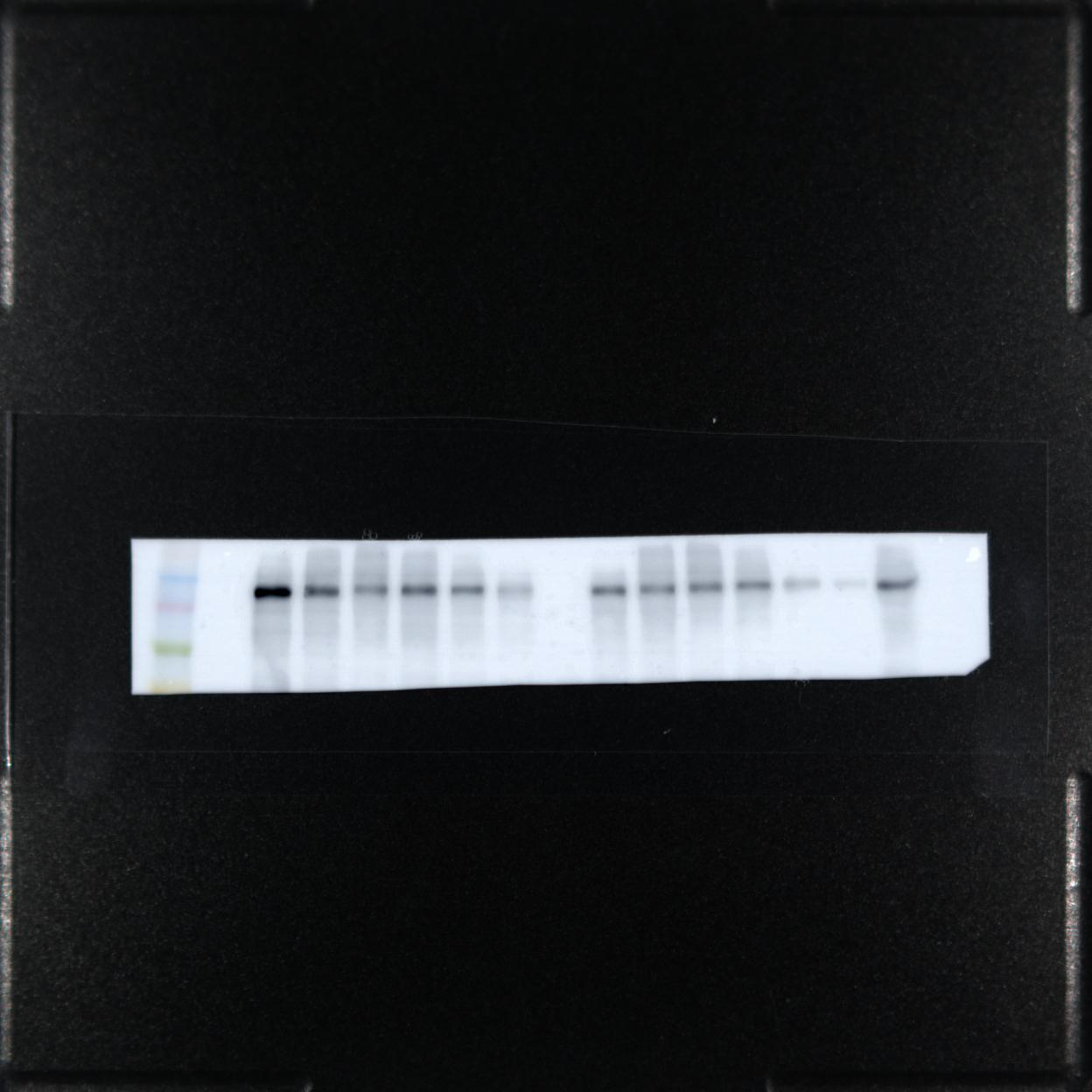

Supplement: Appendix 1—figure 10—source data 2. [file elife-104432-app1-fig10-data2.zip › Appendix Figure 10_Source data 2/Repeat3_pEGFR-1.jpg]

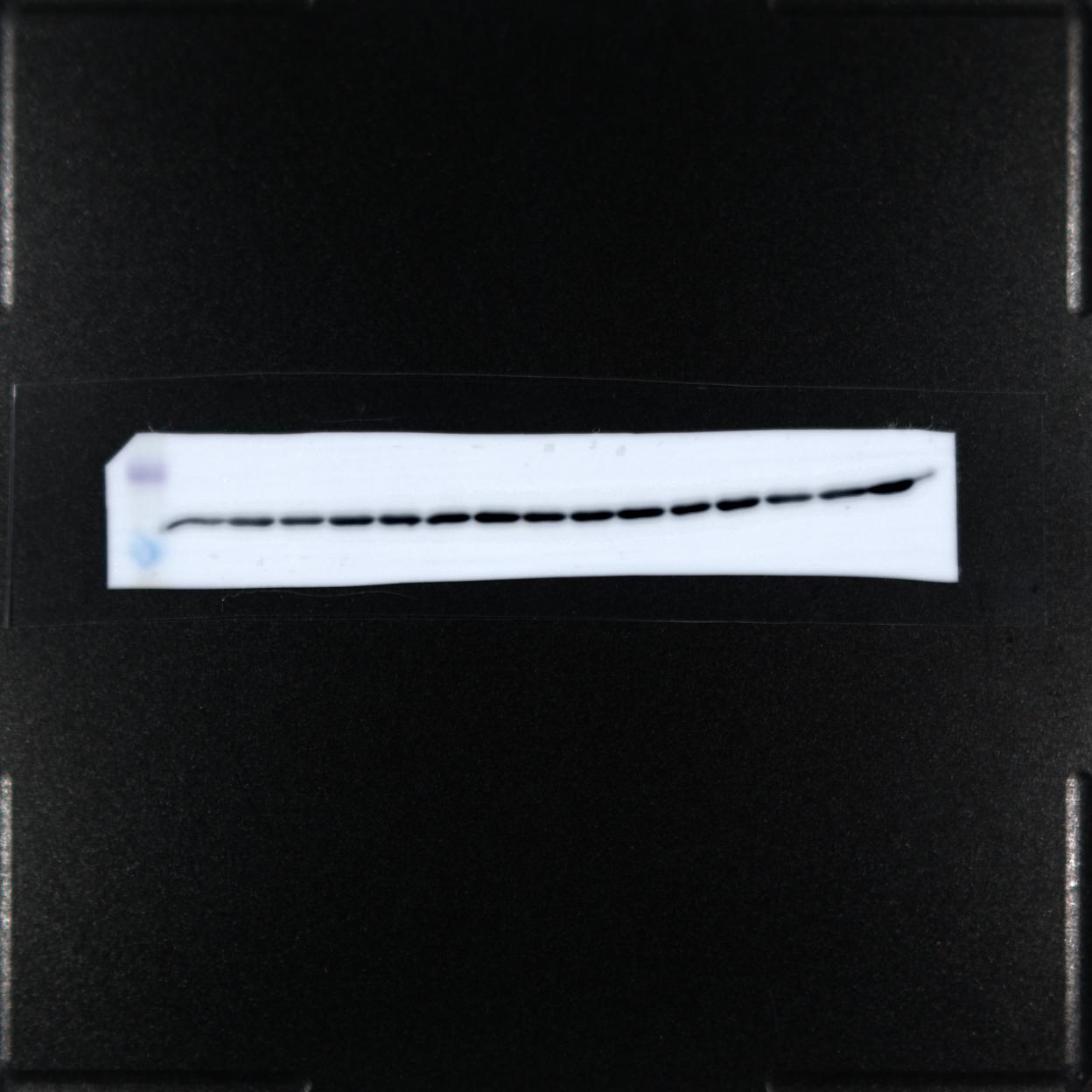

Supplement: Appendix 1—figure 10—source data 2. [file elife-104432-app1-fig10-data2.zip › Appendix Figure 10_Source data 2/Repeat2_actin-1.jpg]

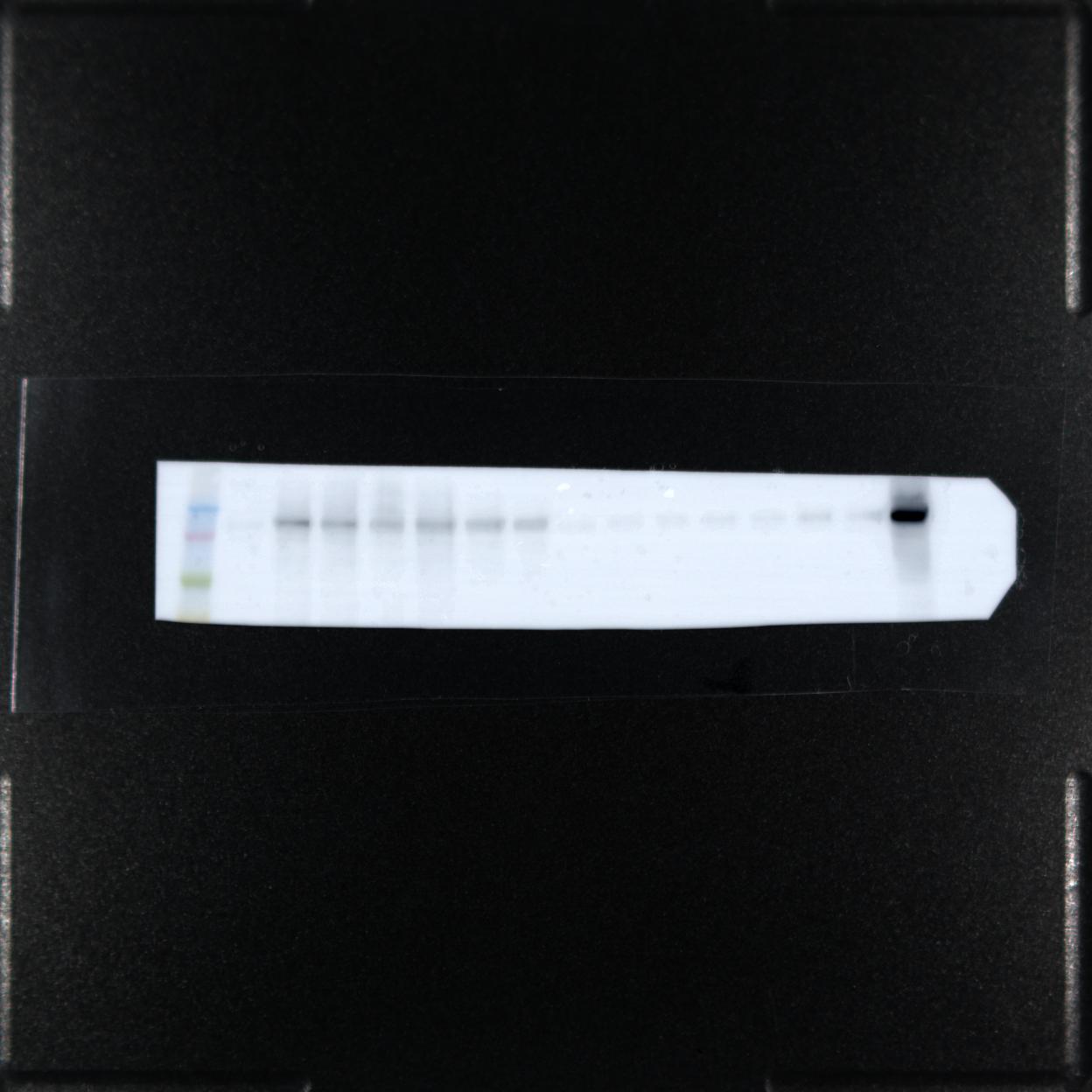

Supplement: Appendix 1—figure 10—source data 2. [file elife-104432-app1-fig10-data2.zip › Appendix Figure 10_Source data 2/Repeat3_pEGFR-2.jpg]

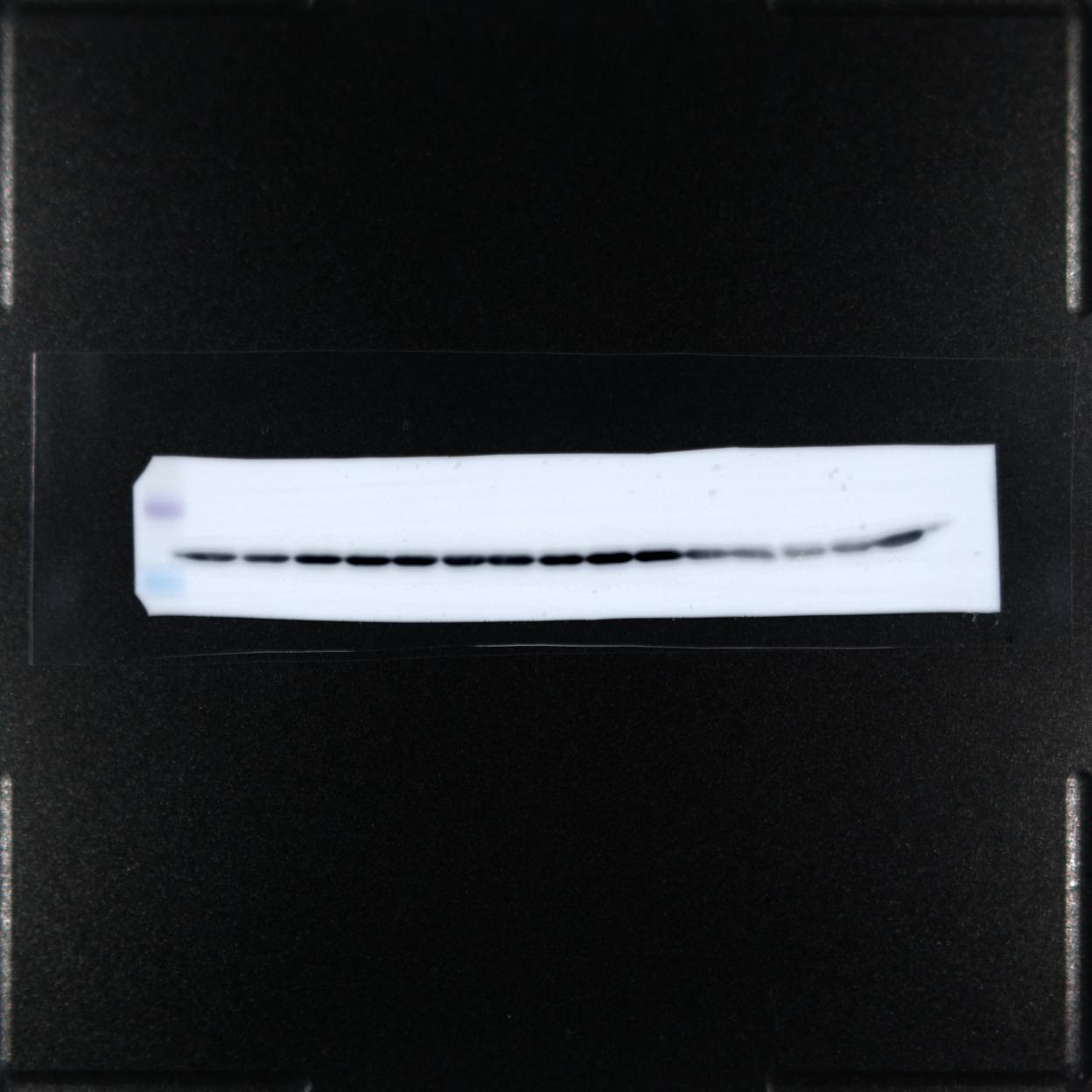

Supplement: Appendix 1—figure 10—source data 2. [file elife-104432-app1-fig10-data2.zip › Appendix Figure 10_Source data 2/Repeat2_actin-2.jpg]

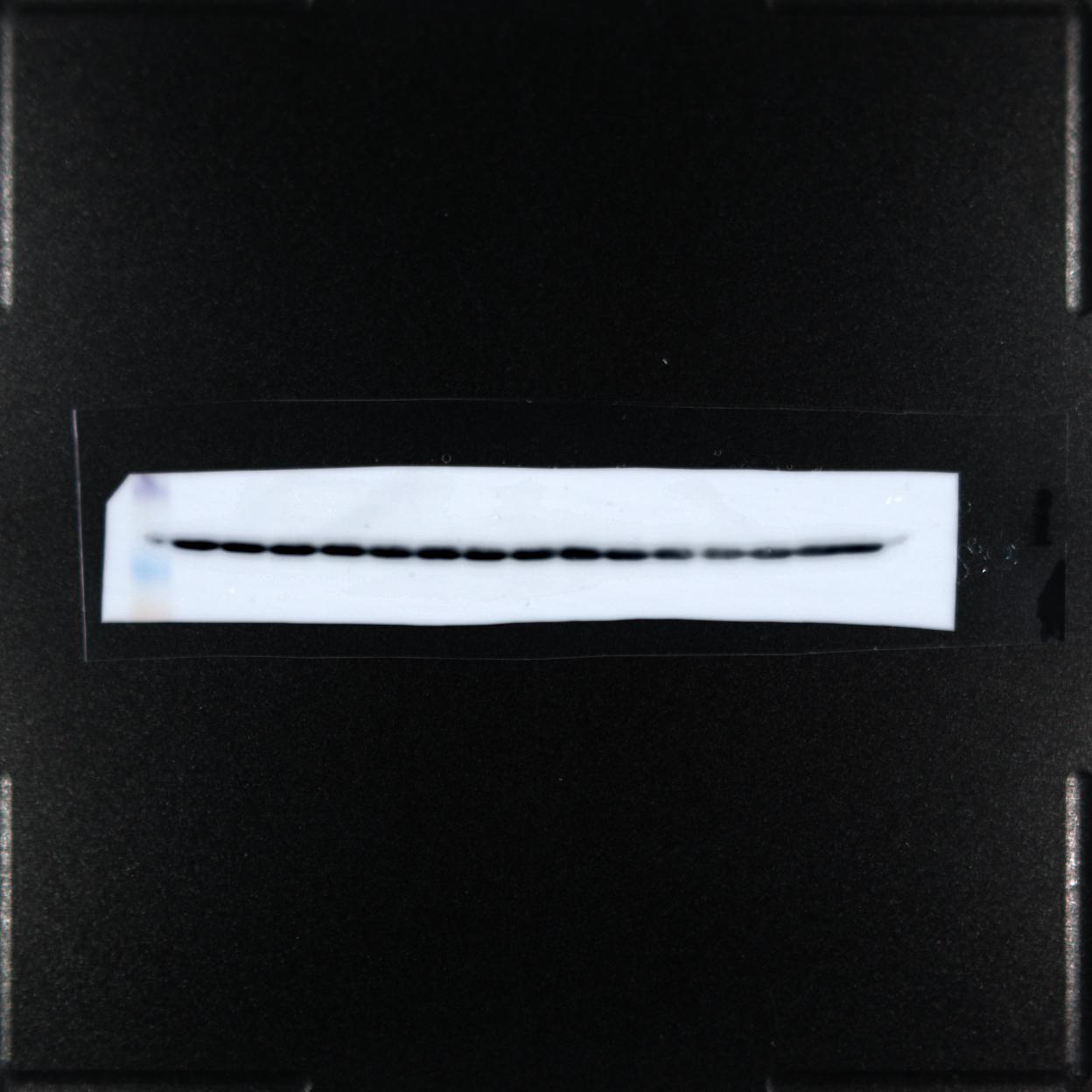

Supplement: Appendix 1—figure 10—source data 2. [file elife-104432-app1-fig10-data2.zip › Appendix Figure 10_Source data 2/Repeat4_actin-1.jpg]

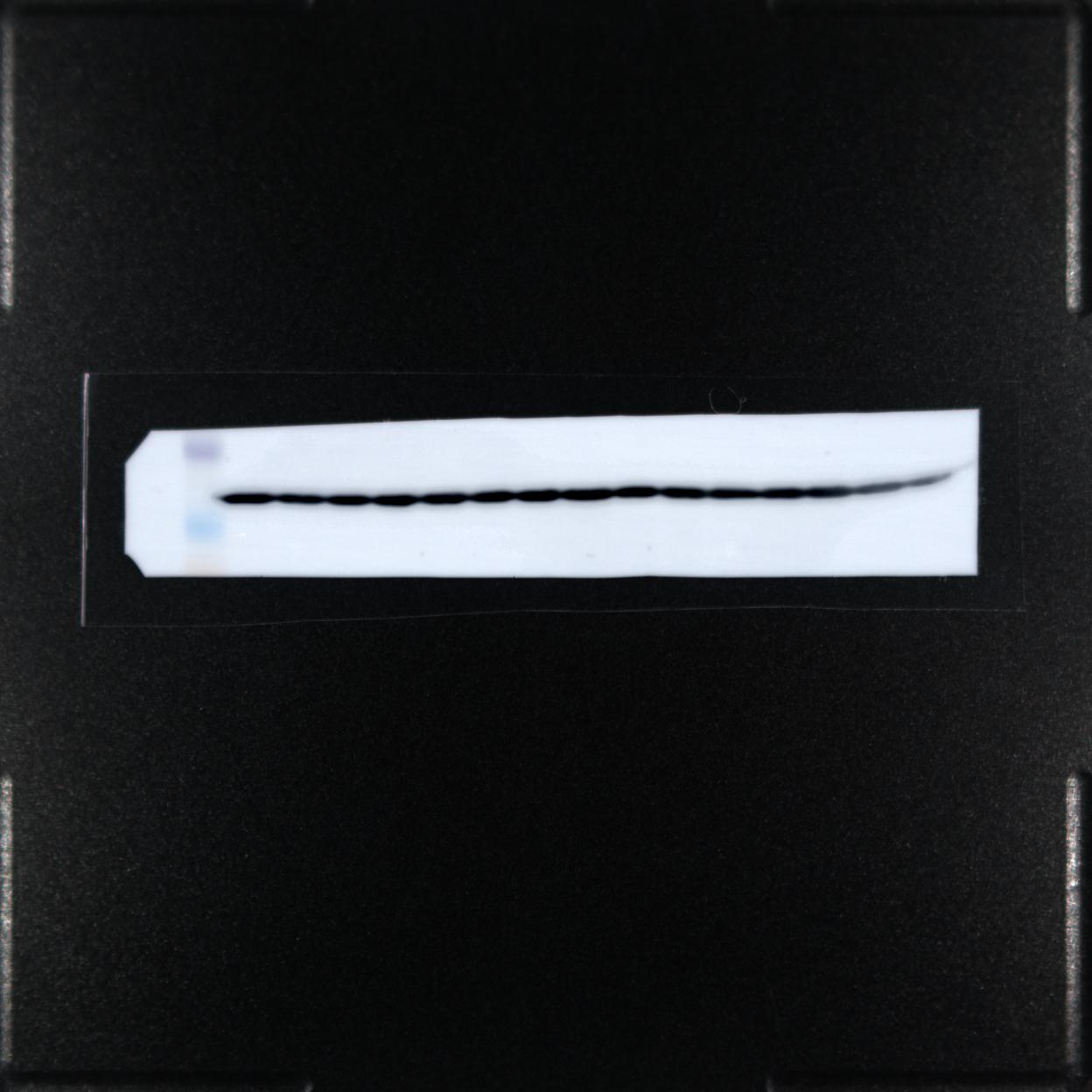

Supplement: Appendix 1—figure 10—source data 2. [file elife-104432-app1-fig10-data2.zip › Appendix Figure 10_Source data 2/Repeat4_actin-2.jpg]

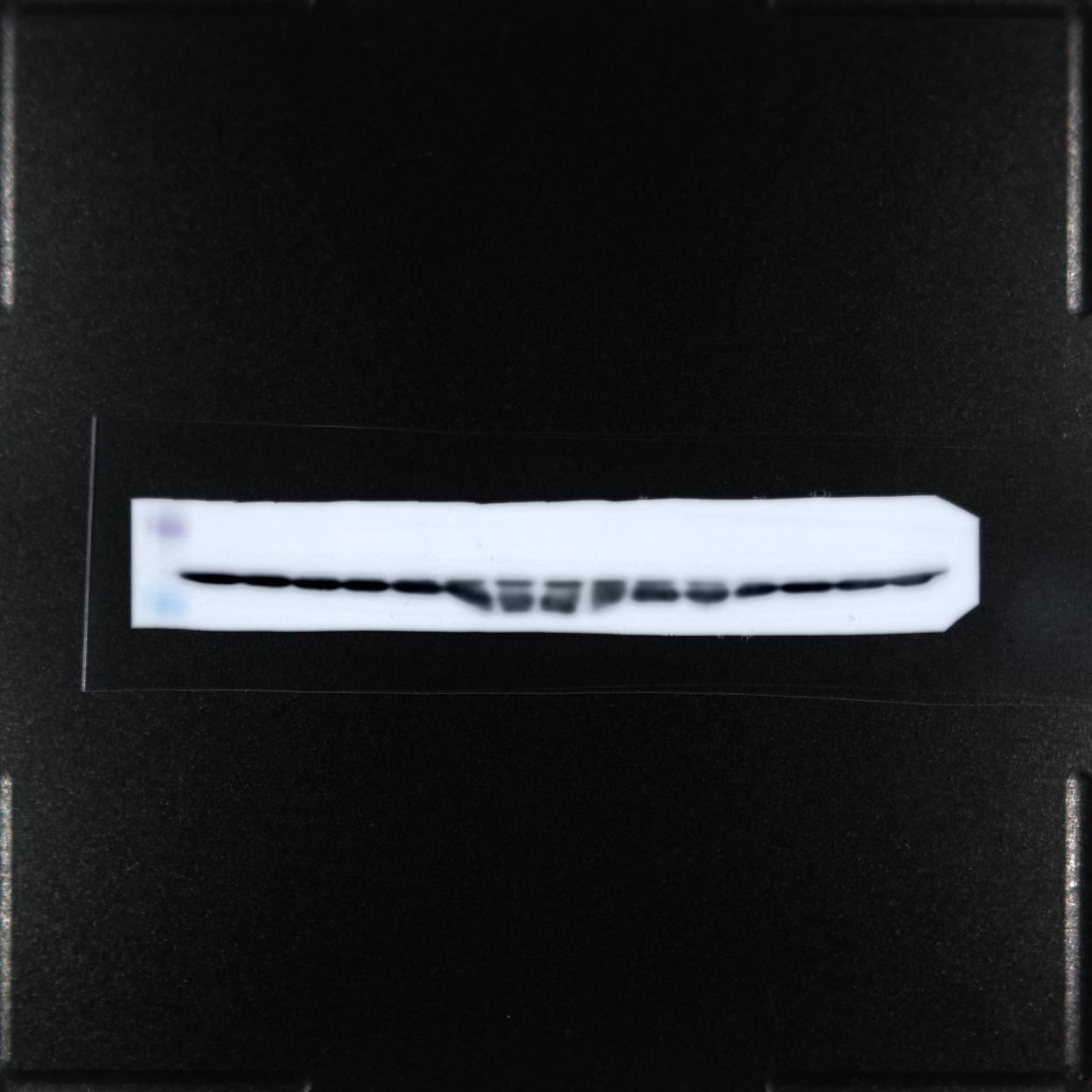

Supplement: Appendix 1—figure 10—source data 2. [file elife-104432-app1-fig10-data2.zip › Appendix Figure 10_Source data 2/Repeat1_actin-2.jpg]

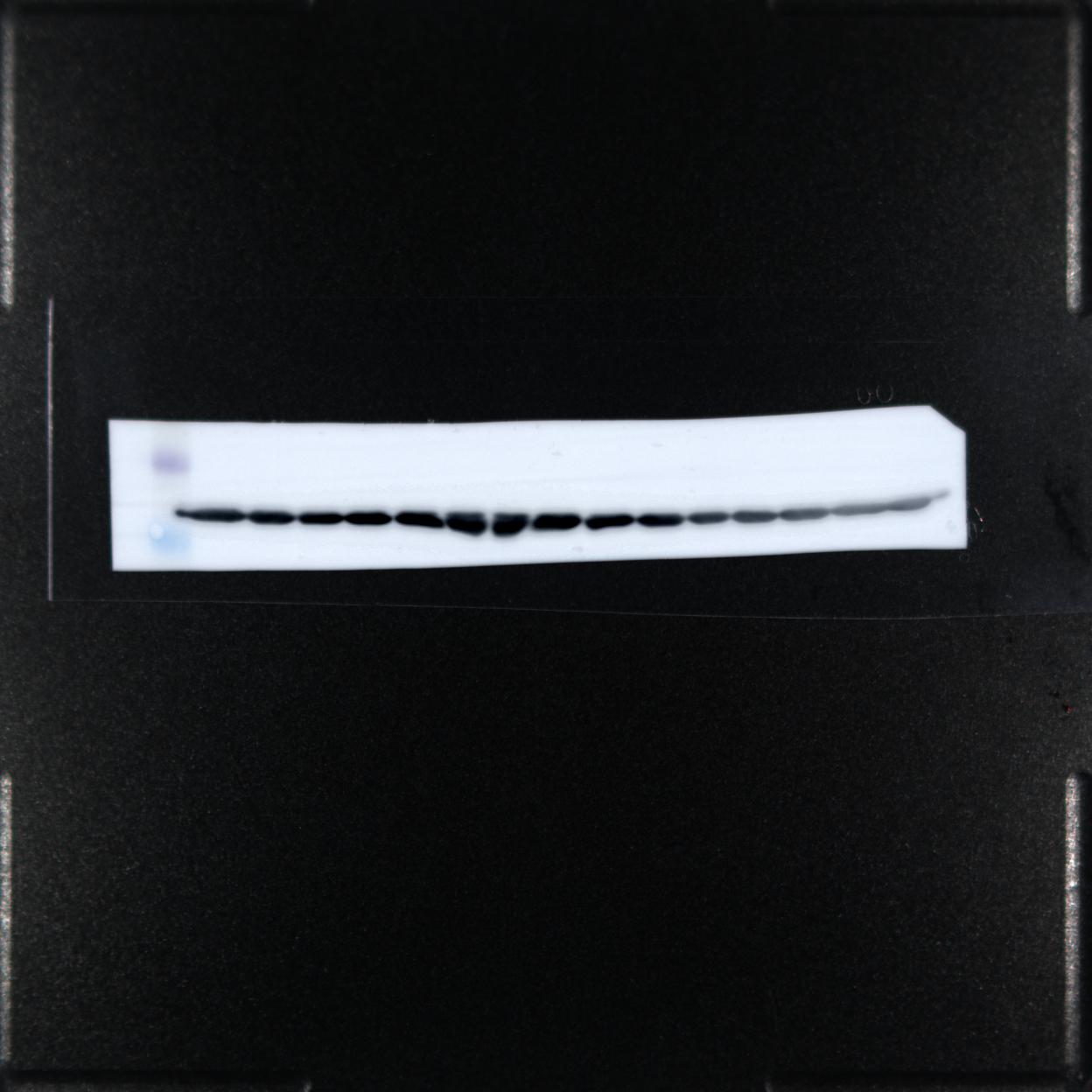

Supplement: Appendix 1—figure 10—source data 2. [file elife-104432-app1-fig10-data2.zip › Appendix Figure 10_Source data 2/Repeat1_actin-1.jpg]

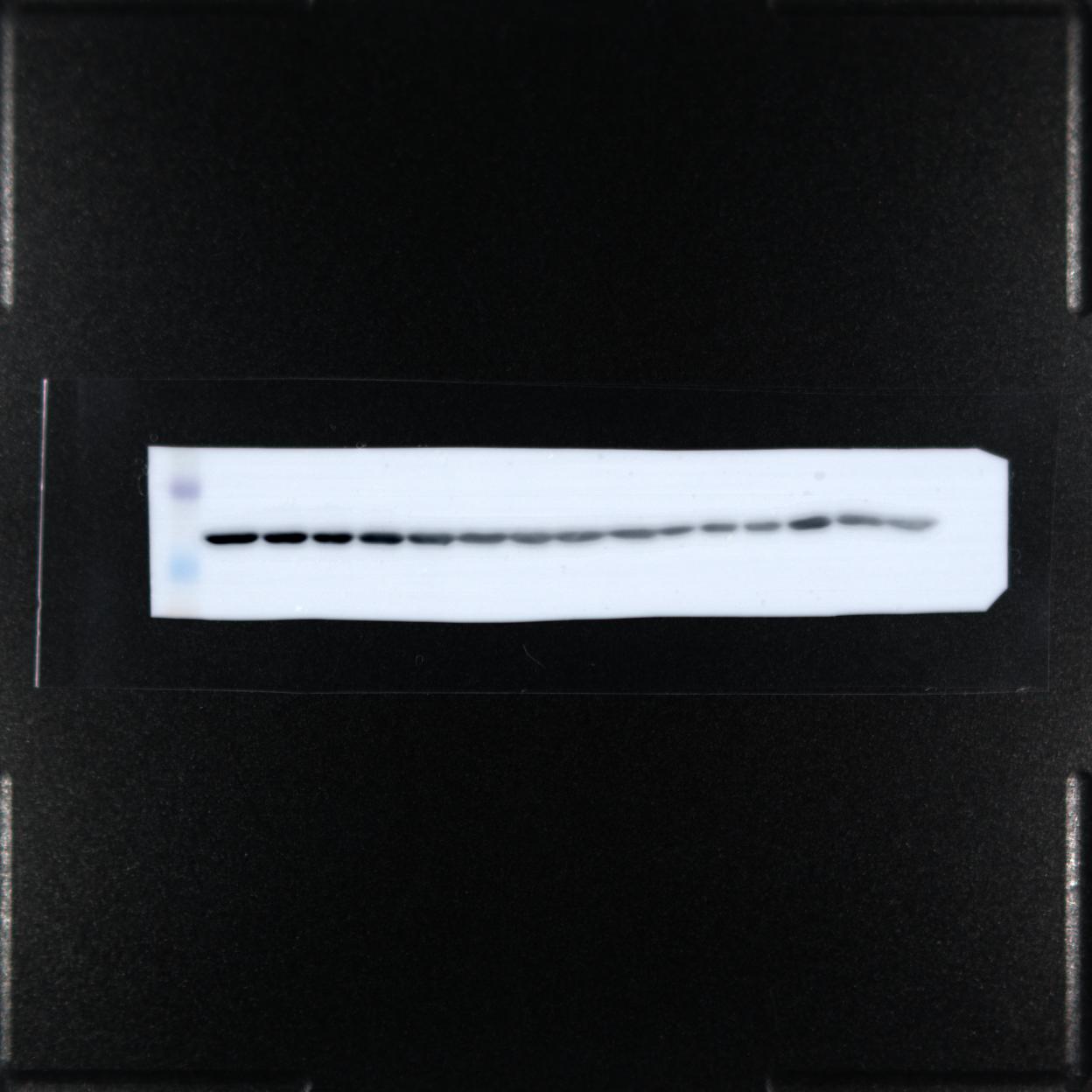

Supplement: Appendix 1—figure 10—source data 2. [file elife-104432-app1-fig10-data2.zip › Appendix Figure 10_Source data 2/Repeat3_actin-2.jpg]

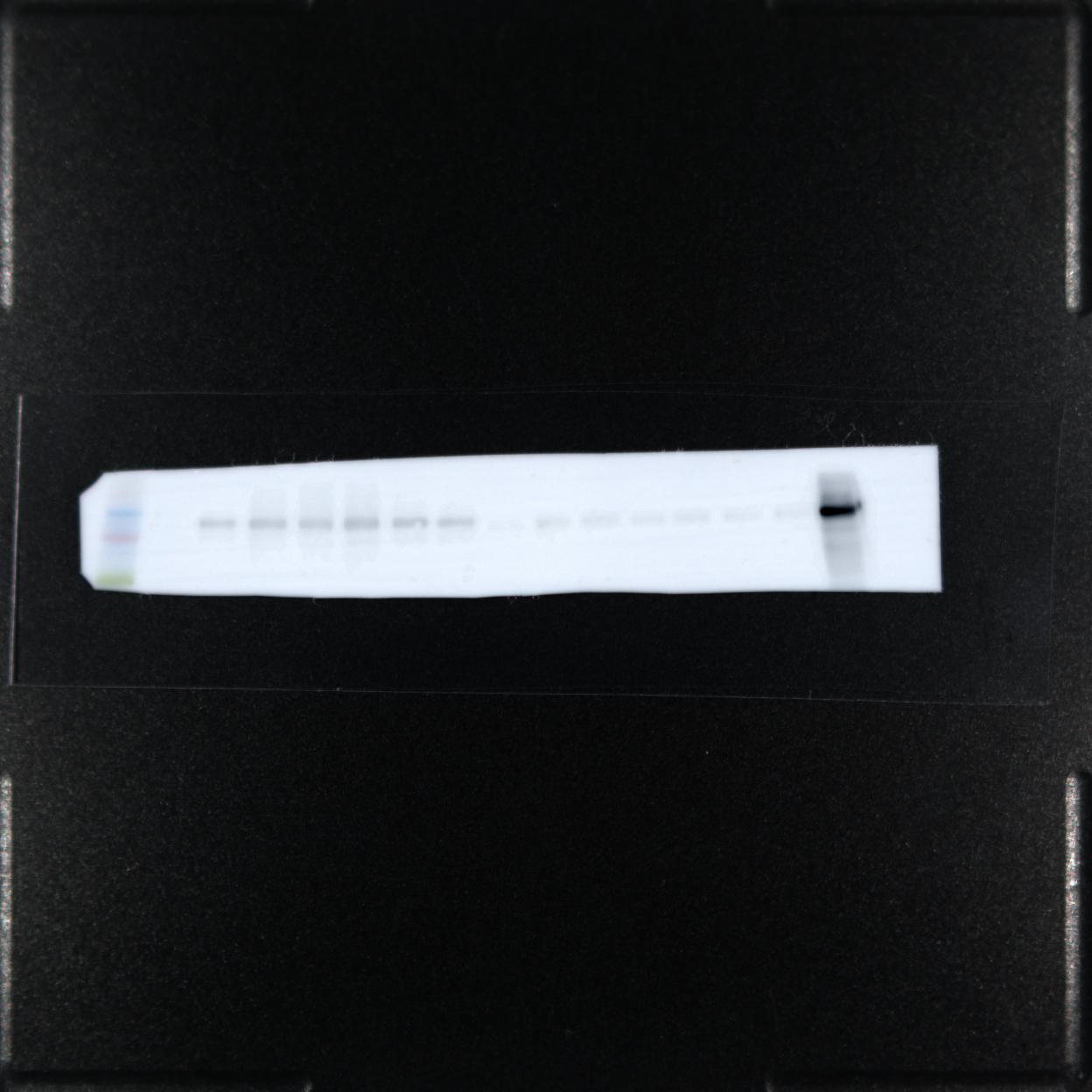

Supplement: Appendix 1—figure 10—source data 2. [file elife-104432-app1-fig10-data2.zip › Appendix Figure 10_Source data 2/Repeat2_pEGFR-2.jpg]

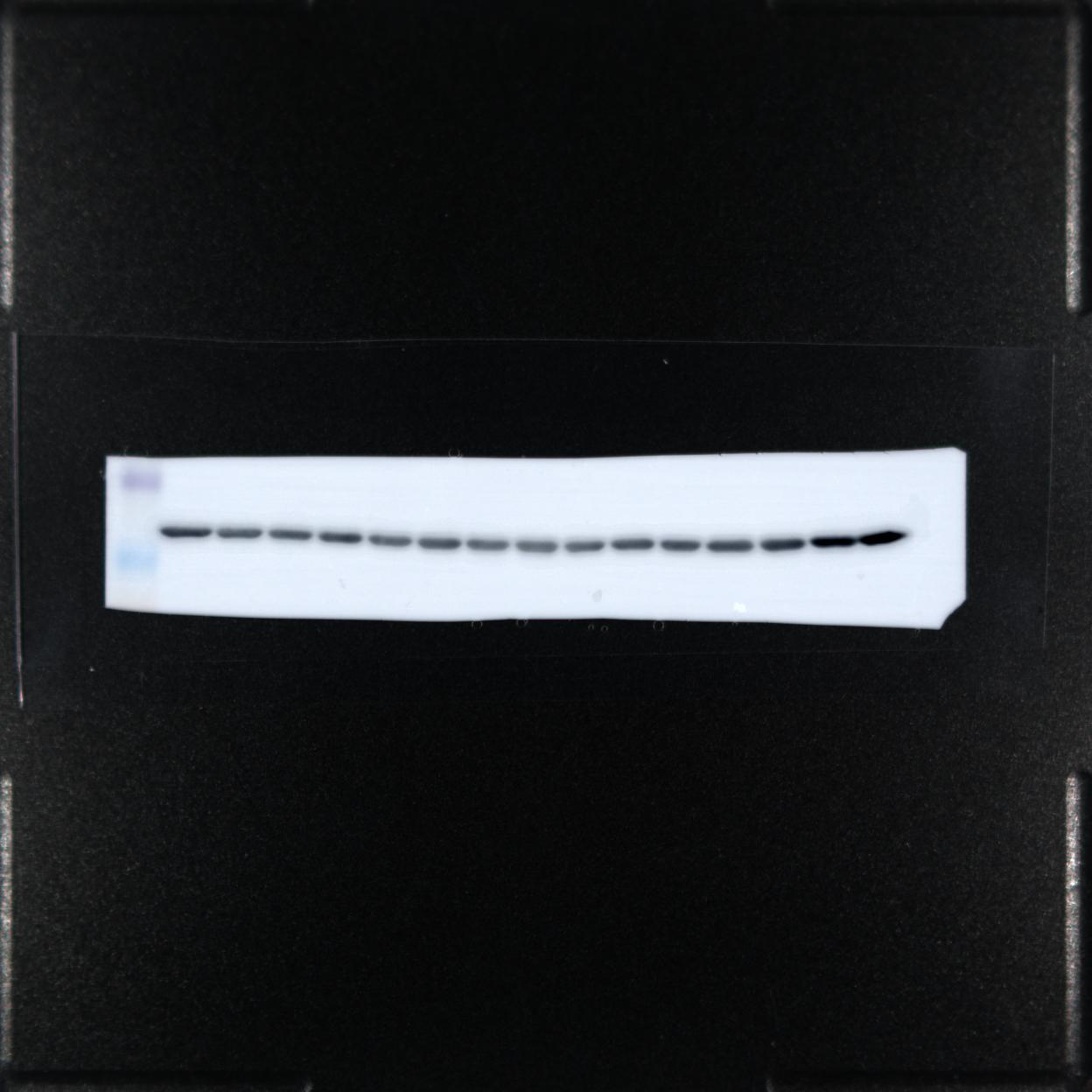

Supplement: Appendix 1—figure 10—source data 2. [file elife-104432-app1-fig10-data2.zip › Appendix Figure 10_Source data 2/Repeat3_actin-1.jpg]

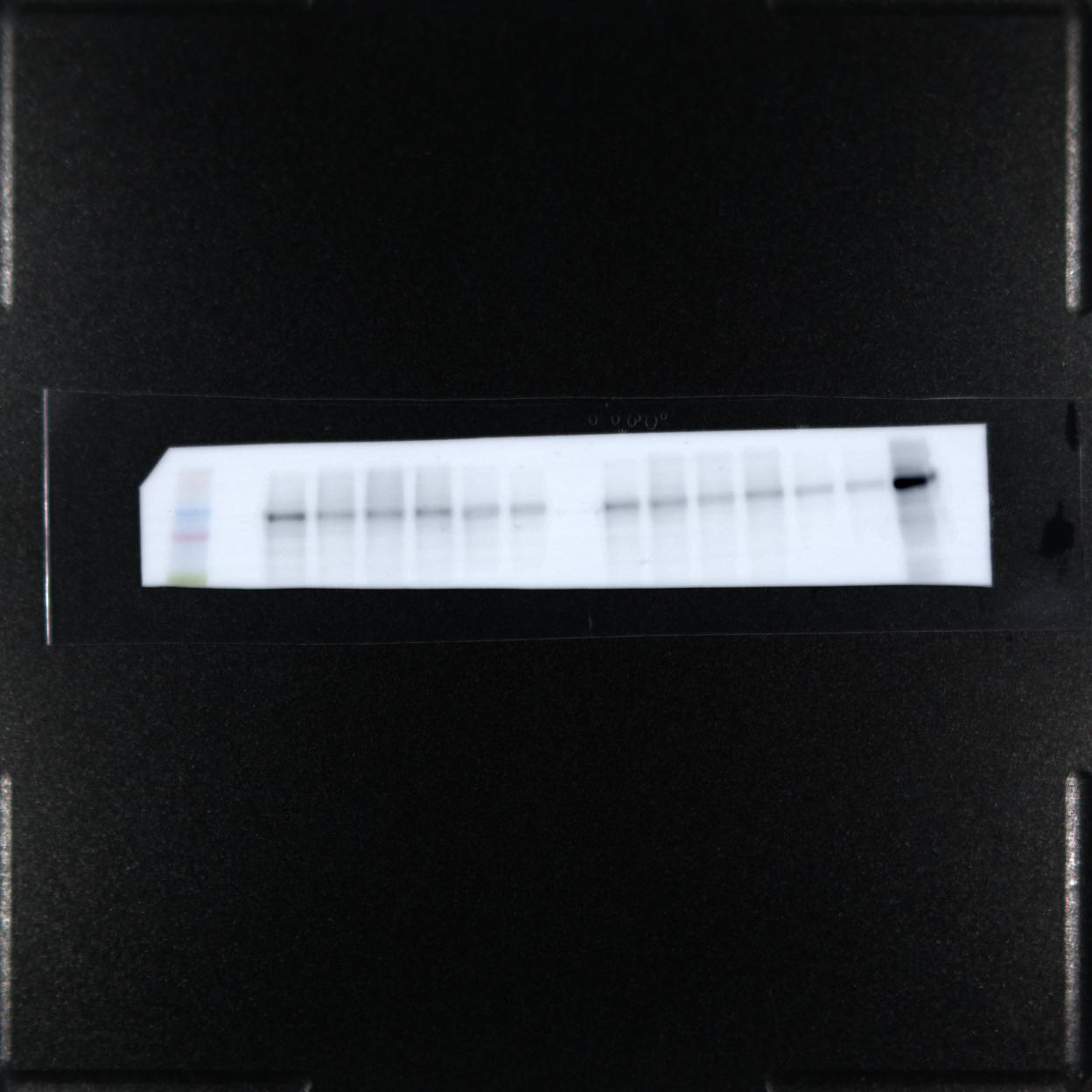

Supplement: Appendix 1—figure 10—source data 2. [file elife-104432-app1-fig10-data2.zip › Appendix Figure 10_Source data 2/Repeat2_pEGFR-1.jpg]

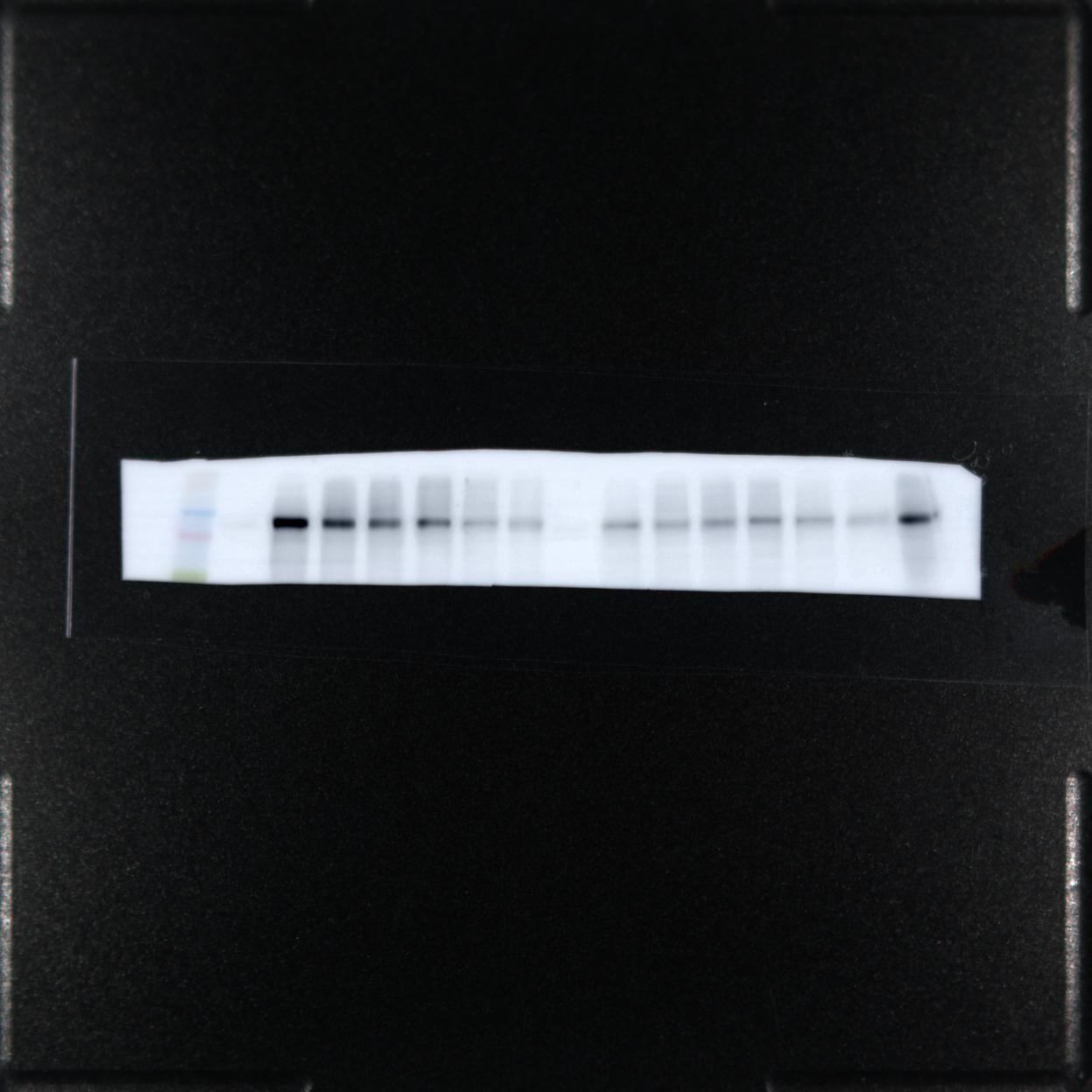

Supplement: Appendix 1—figure 10—source data 2. [file elife-104432-app1-fig10-data2.zip › Appendix Figure 10_Source data 2/Repeat1_pEGFR-1.jpg]

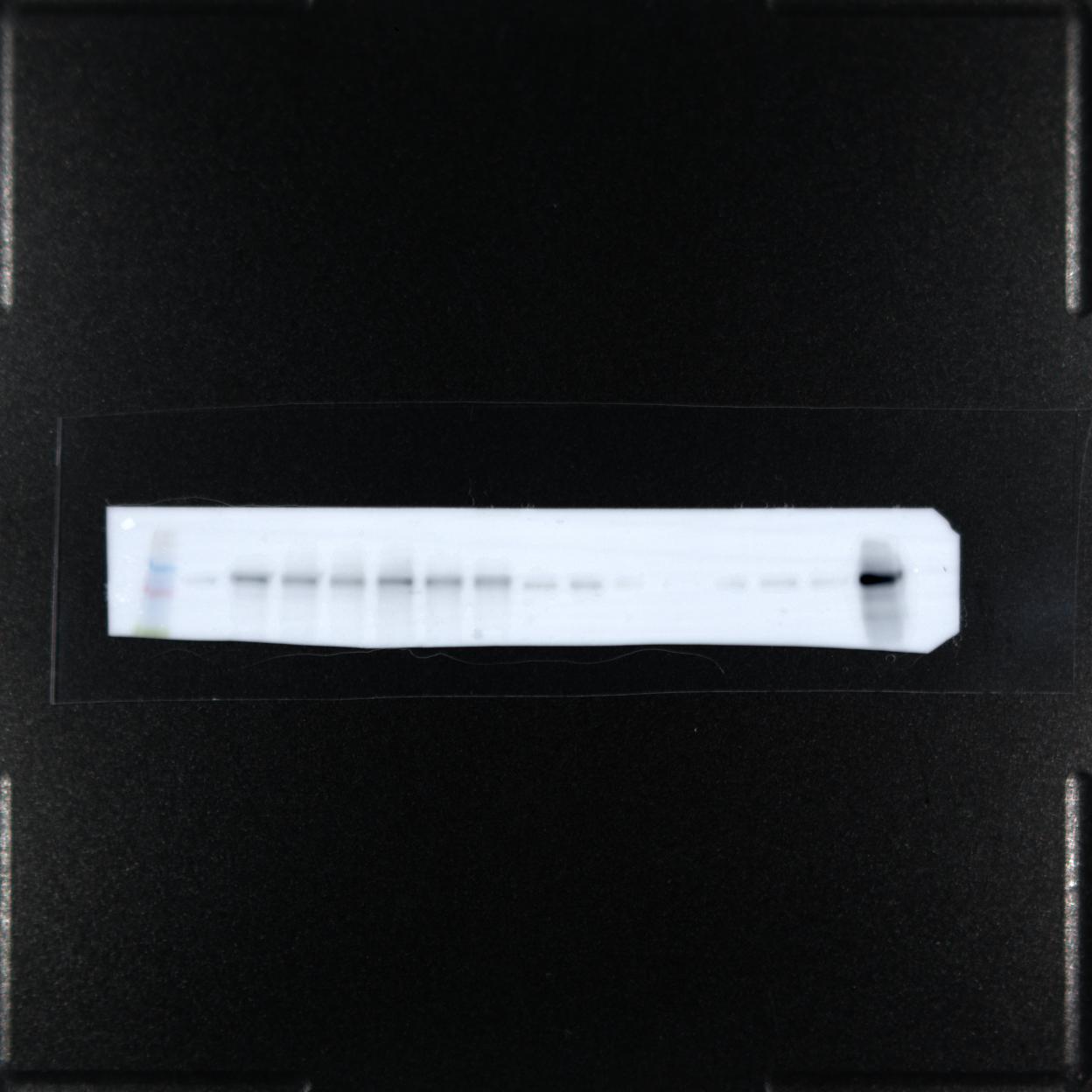

Supplement: Appendix 1—figure 10—source data 2. [file elife-104432-app1-fig10-data2.zip › Appendix Figure 10_Source data 2/Repeat1_pEGFR-2.jpg]

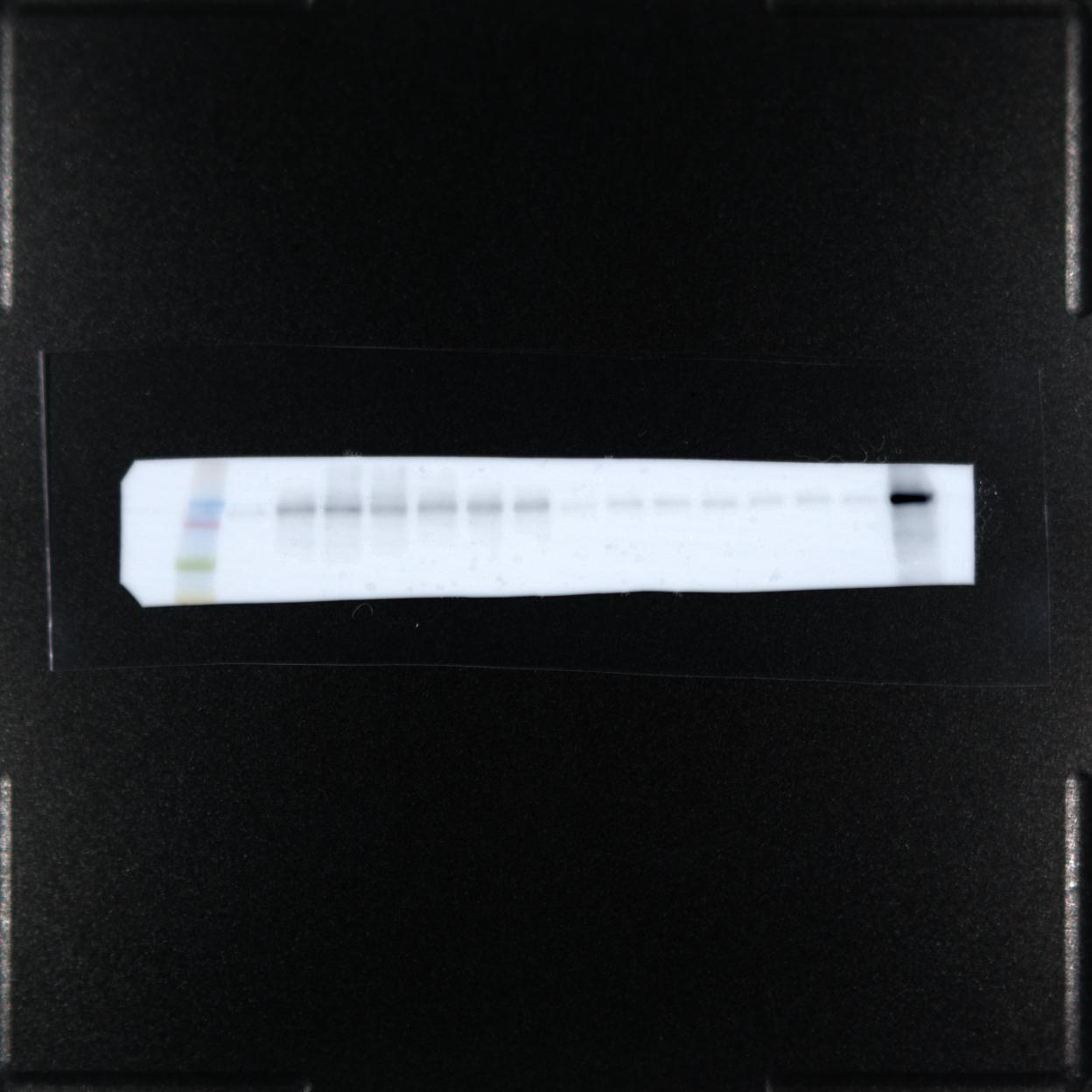

Supplement: Appendix 1—figure 10—source data 2. [file elife-104432-app1-fig10-data2.zip › Appendix Figure 10_Source data 2/Repeat4_pEGFR-2.jpg]

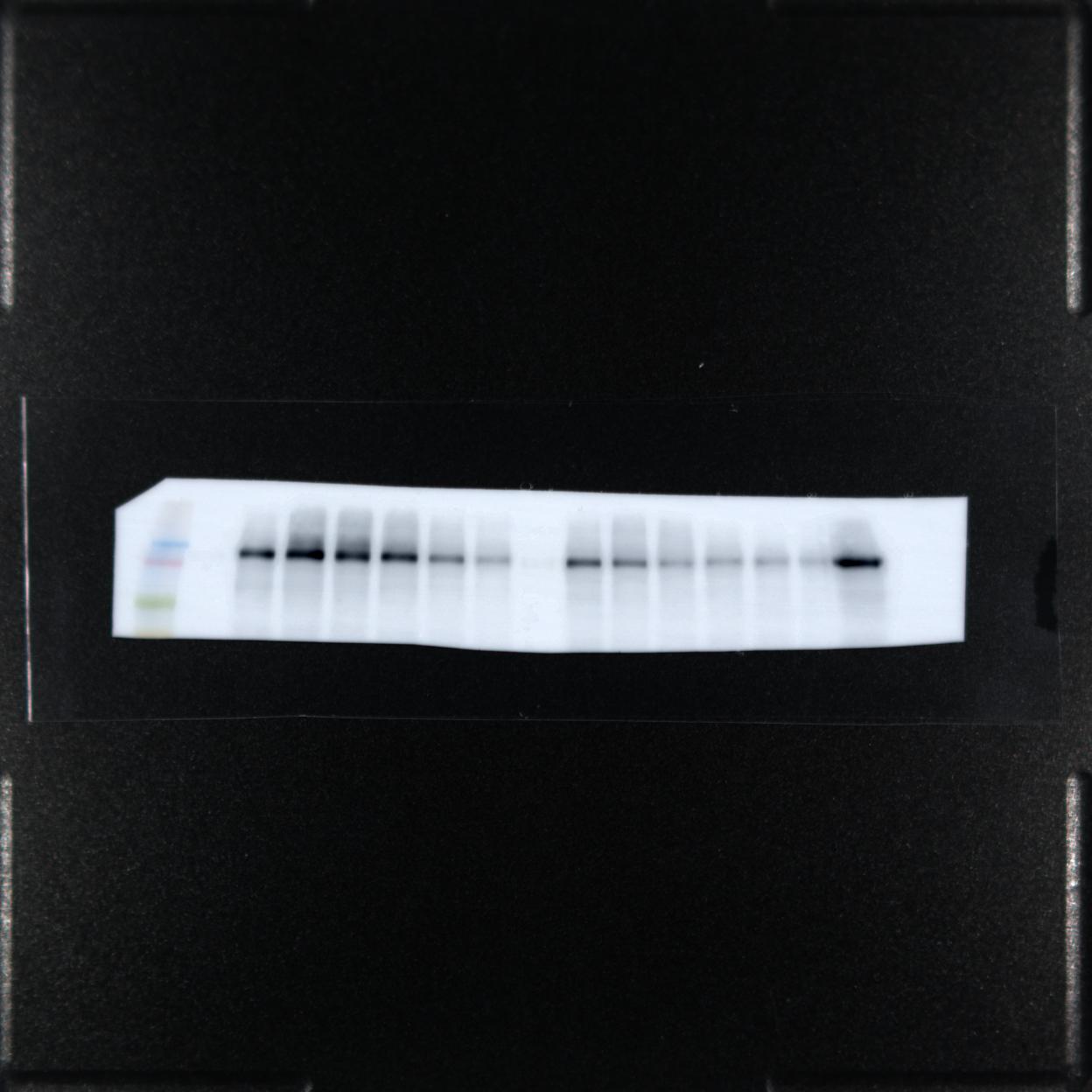

Supplement: Appendix 1—figure 10—source data 2. [file elife-104432-app1-fig10-data2.zip › Appendix Figure 10_Source data 2/Repeat4_pEGFR-1.jpg]
