# Supplementary material for: Evaluation of information flows in the RAS-MAPK system using transfer entropy measurements
Source: eLife. 2025 Mar 6;14:e104432. doi: 10.7554/eLife.104432 (PMC11884788; doi:10.7554/eLife.104432)
Supplement: Figure 5—source data 1. [file elife-104432-fig5-data1.zip › Figure 5_Source data 1.pdf]

Repeat 1

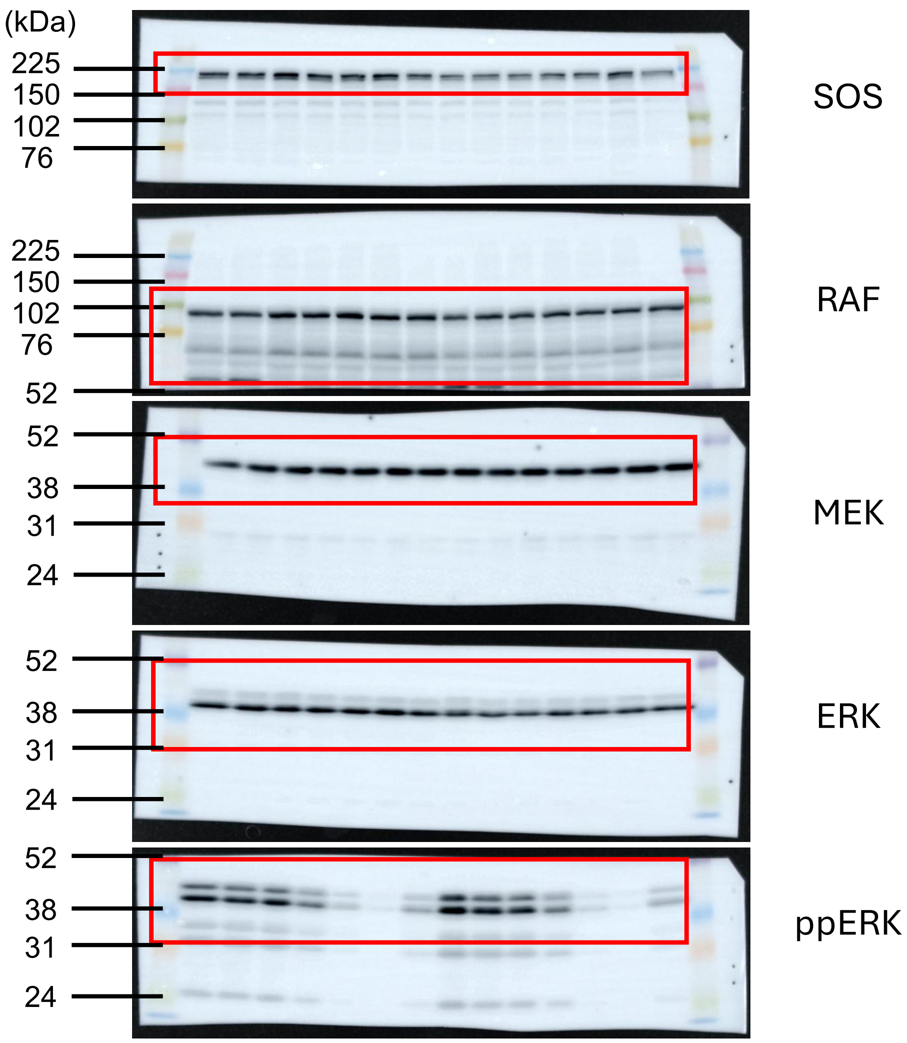

Figure 5a -source data 1-1

Repeat 2

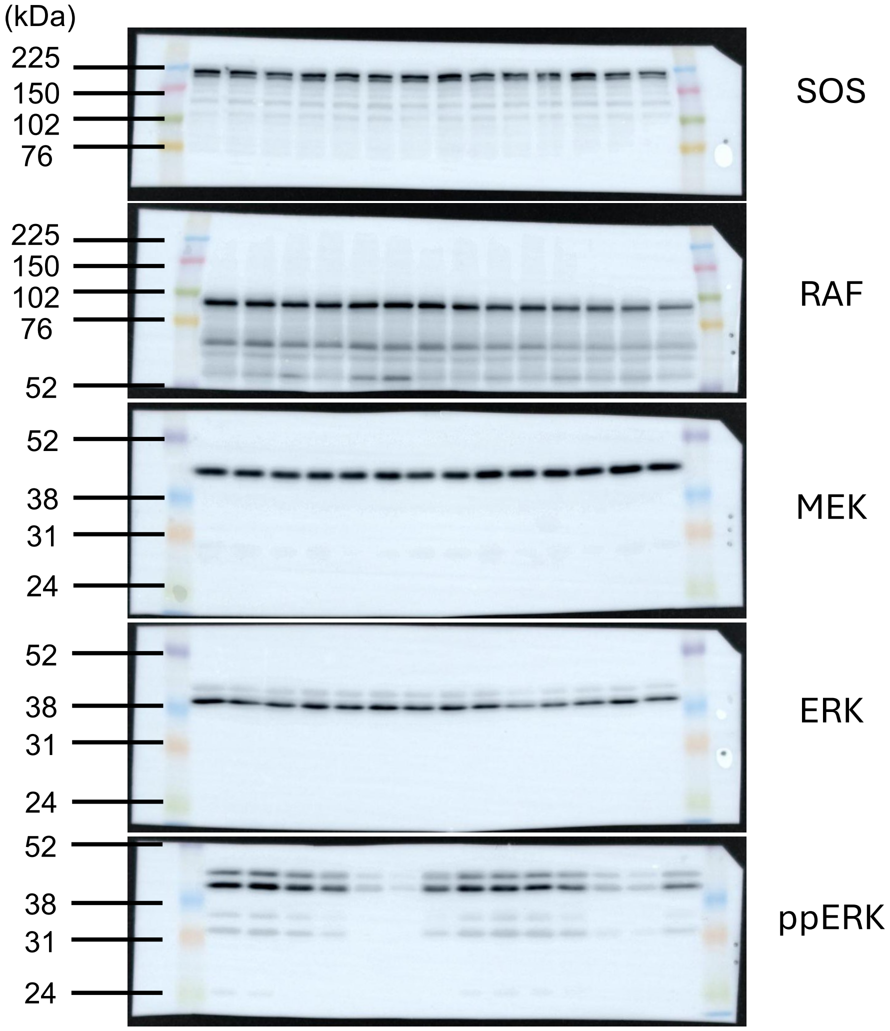

Figure 5a -source data 1-2

Repeat 3

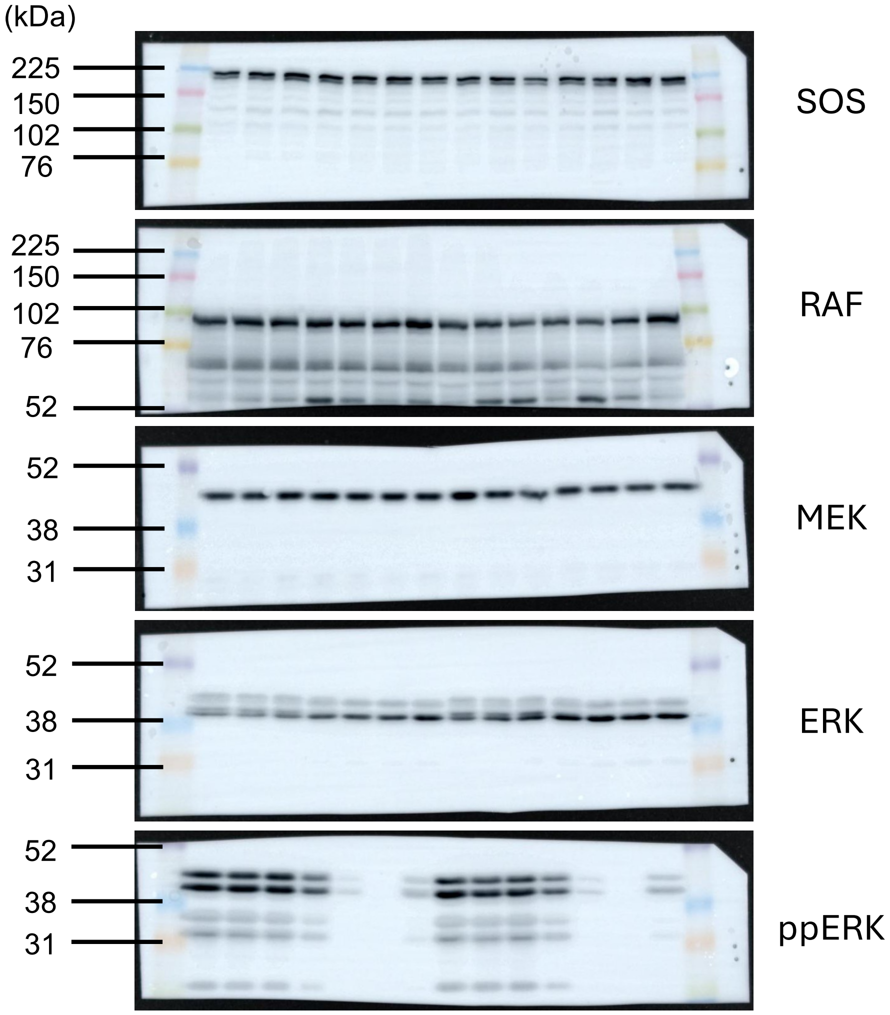

Figure 5a -source data 1-3

Repeat 4

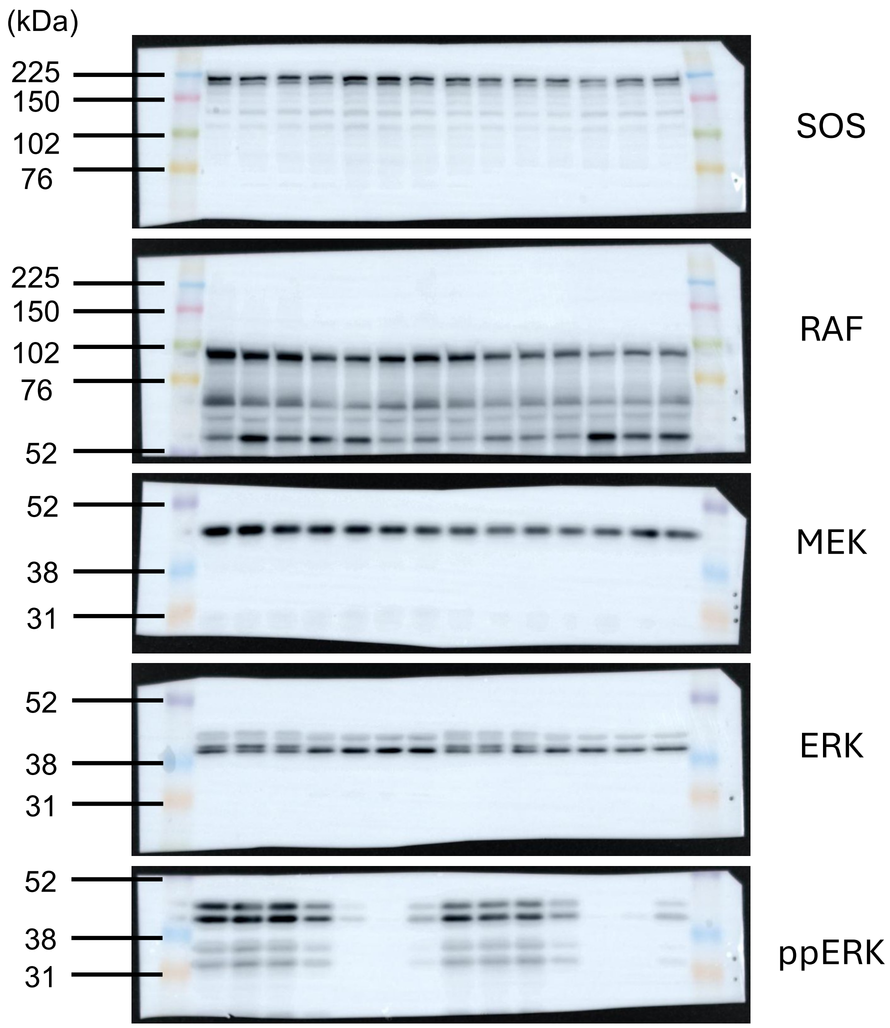

Figure 5a -source data 1-4
